# Biodegradable hollow mesoporous organosilica nanotheranostics (HMON) for multi-mode imaging and mild photo-therapeutic-induced mitochondrial damage on gastric cancer

**DOI:** 10.1186/s12951-020-00653-y

**Published:** 2020-07-20

**Authors:** Weihong Guo, Zhian Chen, Jiajia Chen, Xiaoli Feng, Yang Yang, Huilin Huang, Yanrui Liang, Guodong Shen, Yu Liang, Chao Peng, Yanbing Li, Guoxin Li, Wenhua Huang, Bingxia Zhao, Yanfeng Hu

**Affiliations:** 1 Department of General Surgery, Nanfang Hospital, Southern Medical University, Guangzhou, 510515 China; 2 National Key Discipline of Human Anatomy, School of Basic Medical Sciences, Southern Medical University, Guangzhou, 510000 China; 3 Guangdong Provincial Stomatology Hospital, Southern Medical University, Guangzhou, 510000 China; 4 Department of Medicine Ultrasonics, Nanfang Hospital, Southern Medical University, Guangzhou, 510515 China; 5 Guangdong Provincial Key Laboratory of Cancer Immunotherapy, Guangzhou Key Laboratory of Tumor Immunology Research, Cancer Research Institute, School of Basic Medical Sciences, Southern Medical University, Guangzhou, 510515 People’s Republic of China

**Keywords:** Hollow mesoporous organosilica nanoparticles (HMON), Photo-thermal therapy (PTT), Photo-dynamic therapy (PDT), Multi-modal imaging, Mitochondrial damage

## Abstract

**Background:**

CuS-modified hollow mesoporous organosilica nanoparticles (HMON@CuS) have been preferred as non-invasive treatment for cancer, as near infrared (NIR)-induced photo-thermal effect (PTT) and/or photo-dynamic effect (PDT) could increase cancer cells’ apoptosis. However, the certain role of HMON@CuS-produced-PTT&PDT inducing gastric cancer (GC) cells’ mitochondrial damage, remained unclear. Moreover, theranostic efficiency of HMON@CuS might be well improved by applying multi-modal imaging, which could offer an optimal therapeutic region and time window. Herein, new nanotheranostics agents were reported by Gd doped HMON decorated by CuS nanocrystals (called HMON@CuS/Gd).

**Results:**

HMON@CuS/Gd exhibited appropriate size distribution, good biocompatibility, l-Glutathione (GSH) responsive degradable properties, high photo-thermal conversion efficiency (82.4%) and a simultaneous reactive oxygen species (ROS) generation effect. Meanwhile, HMON@CuS/Gd could efficiently enter GC cells, induce combined mild PTT (43–45 °C) and PDT under mild NIR power density (0.8 W/cm^2^). Surprisingly, it was found that PTT might not be the only factor of cell apoptosis, as ROS induced by PDT also seemed playing an essential role. The NIR-induced ROS could attack mitochondrial transmembrane potentials (MTPs), then promote mitochondrial reactive oxygen species (mitoROS) production. Meanwhile, mitochondrial damage dramatically changed the expression of anti-apoptotic protein (Bcl-2) and pro-apoptotic protein (Bax). Since that, mitochondrial permeability transition pore (mPTP) was opened, followed by inducing more cytochrome c (Cyto C) releasing from mitochondria into cytosol, and finally activated caspase-9/caspase-3-depended cell apoptosis pathway. Our in vivo data also showed that HMON@CuS/Gd exhibited good fluorescence (FL) imaging (wrapping fluorescent agent), enhanced T1 imaging under magnetic resonance imaging (MRI) and infrared thermal (IRT) imaging capacities. Guided by FL/MRI/IRT trimodal imaging, HMON@CuS/Gd could selectively cause mild photo-therapy at cancer region, efficiently inhibit the growth of GC cells without evident systemic toxicity in vivo.

**Conclusion:**

HMON@CuS/Gd could serve as a promising multifunctional nanotheranostic platform and as a cancer photo-therapy agent through inducing mitochondrial dysfunction on GC.

## Background

Gastric cancer (GC) is one of the most common malignant tumors and its mortality rate ranks third worldwide. Despite of the great progress of traditional chemotherapy and molecular target therapy, the prognosis of GC is still relatively poor, especially in China [1, 2]. Considering the limitations of current diagnosis and therapy methods, there is a pressing need to identify novel potential strategies to offer new, improved diagnosis and therapy methods for GC.

Recently, reactive oxygen species (ROS) have attracted more attention due to its regulation in cancer development. Moderate levels of ROS could promote tumor progression by inducing DNA mutations, genomic instabilities or acting as signaling molecules that accelerate cancer cell proliferation and metastasis [3]. In contrast, excessive levels of ROS might enhance cellular oxidative stress, cause DNA/proteins/lipids damage, and lead to apoptotic cell death [4]. As the main organelle for ROS production, mitochondria are often the victim of exogenous elevated ROS exposure with deadly consequences, such as oxidative damage of mitochondrial DNA (mitoDNA), oxidative respiratory chain and mitochondrial membrane permeability [5, 6]. To our knowledge, mitochondria play a significant role in numerous biological processes, including the initiation of cell death, cellular energy generation and metabolic integration [7, 8]. Accumulating evidence has identified that mitochondrial dysfunction was always accompanied with interfered oxidative respiratory chain, which might reduce intracellular ATP levels and fail to produce enough energy for tumor growth [9, 10]. Meanwhile, mitochondrial damage induces cytochrome C (Cyto C) leakage from mitochondria into cytosol, while high levels of Cyto C subsequently activate caspase-depended apoptosis pathway [11, 12]. Therefore, boosting ROS could be a crucial way for activating mitochondria-depended apoptosis pathway, while precise ROS-generation at GC region is still a big problem.

Near infrared light (NIR)-based photo-therapy (PT) has been preferred for tumors targeting treatment, due to the advantages of minimal harm to normal tissues, non-invasiveness, and efficient therapeutic abilities [13, 14]. Emerging studies have also identified that with the assistance of NIR, photosensitizers could increase the production of ROS to cause mitochondria-depended cancer cell death [15–17]. To some extent, the combination of photo-thermal therapy (PTT) and photo-dynamic therapy (PDT) could achieve much better therapeutic efficiency, as hyperthermia has been reported with the ability to elevate the level of oxygen in the tumor due to the increase of blood flow [18, 19], thus overcoming the hypoxia-associated resistance for PDT. Several hollow mesoporous organosilica nanotheranostics (HMON) carrying CuS, ICG or 7AAG, had been reported with ROS generation ability and intrinsic photo-thermal property under single laser irradiation, which could be used for PDT&PTT synergistic therapy, utilizing the good dispersion and high drug-loading capability of HMON and the photothermal ability of HMON [20, 21].

However, the lasers employed for the PTT&PDT treatments are usually different, thus the time interval between different modes will affect the synergistic efficacy [22]. Meanwhile, since PTT-treated cells readily acquire tolerance to heat stress, a relatively high temperature (> 50 °C) was required to achieve the desired therapeutic effect, which would inevitably cause damage to normal organs near the tumor [23, 24]. Therefore, there is still a great need to provide the synchronous implementation of PDT&PTT utilizing a single laser irradiation at mild-temperatures (43–45 °C), while targeted delivery of a photosensitizers to the intended area holds potential to pinpoint the site of therapeutic efficacy. Furthermore, the certain role of PTT&PDT influencing GC cells’ apoptosis at mild temperature also needs further exploration.

**Scheme 1:**
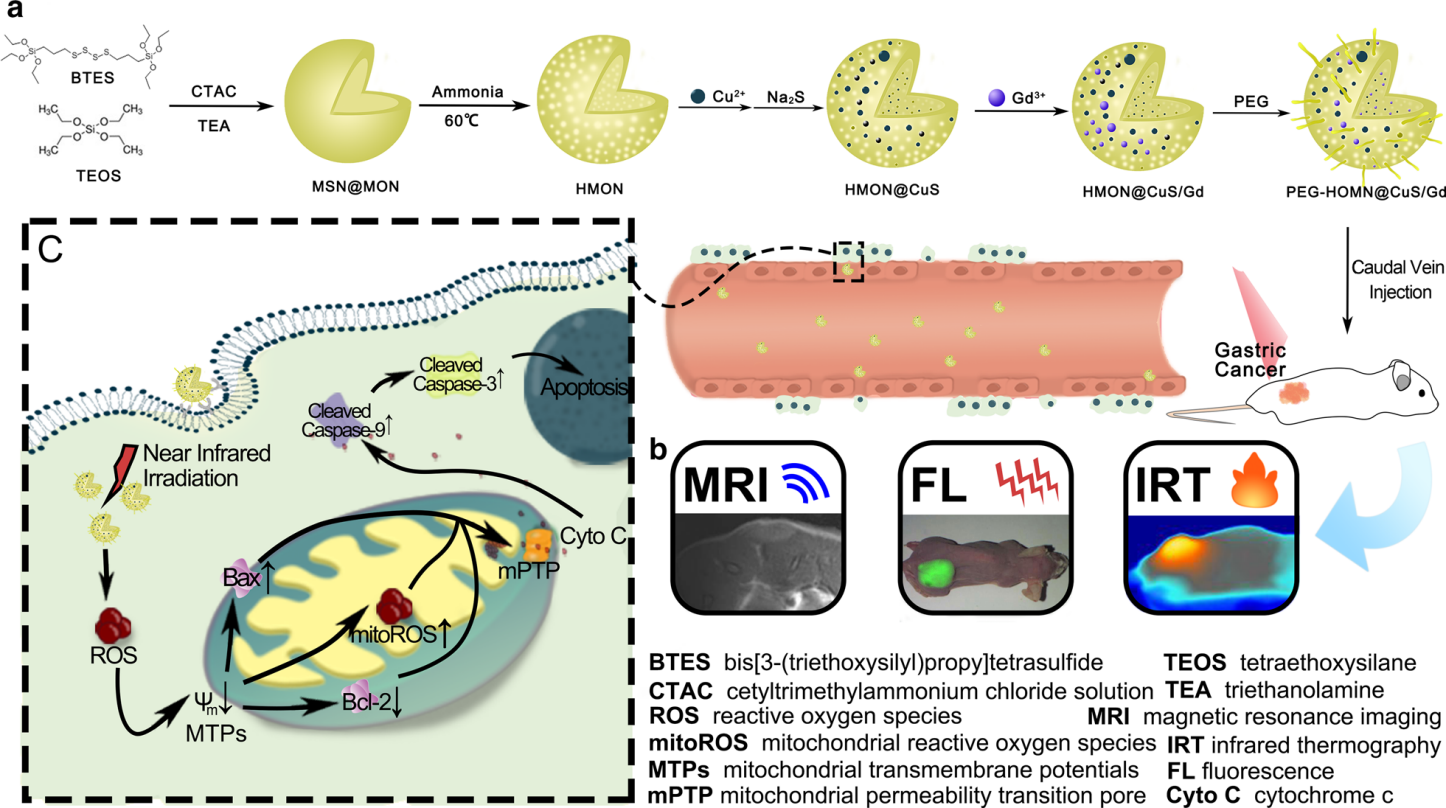
The synthesis process, FL/MRI/IRT trimodal imaging abilities and anti-tumor mechanism of HMON@CuS/Gd in GC cells

Herein, HMON nanotheranostics were decorated with CuS nanocrystals (HMON@CuS NPs) via in situ growth, following the protocol of our previous work where CuS nanocrystals were in situ grown onto the surface of hollow mesoporous nanospheres [19]. The designed nanoplatform performed well PDT and PTT conversion efficiency and better biocompatibility. Furthermore, Gd was doped into the into the hollow structure, while good fluorescence (FL) imaging (wrapping fluorescent agent), enhanced T1 imaging under magnetic resonance imaging (MRI) and infrared thermal (IRT) trimodal imaging could therefore be achieved to guide photo-therapy [25]. After treated with HMON@CuS/Gd under NIR irradiation, in vitro and in vivo GC proliferation abilities were evaluated. Moreover, to fully elucidate whether ROS-mediated mitochondrial apoptosis pathway contributed to the anti-tumor effects induced by HMON@CuS/Gd, we detected the changes of ROS levels and the biological function of mitochondria, including mitochondrial transmembrane potentials (MTPs), mitochondrial ROS (mitoROS) and mitochondrial permeability transition pore (mPTP) produced levels, along with the expression levels of Cyto C and apoptosis-related proteins. Above all, our well designed HMON@CuS/Gd might present a promising approach for the imaging-guided treatment of GC (Scheme 1).

## Materials and methods

### Materials

Cetyltrimethylammonium chloride solution (CTAC), triethanolamine (TEA), tetraethoxysilane (TEOS), sodium citrate (3-mercaptopropyl)-trimethoxysilane (MPTES), concentrated HCl (37%), ammonia aqueous solution (NH_3_·H_2_O, 25wt %), bis[3-(triethoxysilyl)propyl]tetrasulfide (BTES), sodium sulfide nonahydrate (Na_2_S·9H_2_O), copper chloridedihydrate (CuCl_2_·2H_2_O), Gadolinium chloride hexahydrate (GdCl_3_·6H_2_O) were purchased from Sigma-Aldrich (MO, USA). The C18PMH-mPEG was purchased frommLaysan Bio Inc. (AL, USA). The PBS, DMEN medium, Fetal bovine serum (FBS) and 0.05% trypsin–EDTA were obtained from Gibco Laboratories (NY, USA). Human stomach normal epithelial cells (GES-1), human stomach cancer cells (HGC-27), human lung normal epithelial cells (BEAS-2B), primary spleen cells of mice (spleen cells) and human liver normal epithelial cells (LO2) were acquired from American Type Culture Collection (ATCC).

### Synthesis of HMON

In this study, we applied an ammonia-assisted selective etching strategy to construct the hollow structure of HMON nanocarriers. Briefly, 2.1 mL of CTAC solution and 50 ul TEA solution were added into 20 mL deionized water (ddH_2_O), followed by stirred at 95 °C, and then 1 mL of TEOS were dropwised into the mix solution. 1 h later, a mixture of BTES (1 mL) and TEOS (1 mL) were dropwised and reacted for another 4 h to form MSN@MON products. The MSN@MON products were washed with ethanol for three times. Subsequently, the MSN@MON products were suspended with 30 mL ddH_2_O containing 8.4 mL HCl solution (37%) and stirred at 80 °C for 12 h to remove the unreacted CTAC, then washed with ethanol and repeated the reaction once again. After that, a mixture of 20 mL of ddH_2_O and 13.5 mL ammonia solution were added and reacted at 60 °C for another 3 h. Finally, the HMON products were obtained after centrifugation and washing with ddH_2_O for several times.

### Synthesis of HMON@CuS/Gd

Firstly, 30 mg of HMON products were suspended with 40 mL ethanol solution. Subsequently, 15 mg of CuCl_2_·6H_2_O were added and reacted for another 6 h. After washing with ethanol for several times, 30 mg of Na_2_S were added and stirred overnight at room temperature. Following that, 15 mg of GdCl_3_·6H_2_O were added into the obtained solution for 12 h, washed with ethanol for several times, and finally HMON@CuS/Gd were collected after centrifugation. Furthermore, in order to the water-solubility and biocompatibilities, HMON@CuS/Gd were stirred with 15 mg of C18PMH-mPEG [26] and kept stirring for 1 h before blow-drying of the chloroform. Finally, the PEG-modified HMON@CuS/Gd were collected by centrifugation and washed with ddH_2_O for several times.

### Characterization of HMON@CuS/Gd

The surface morphology and elemental mapping images of HMON@CuS/Gd were captured by JEOL JEM-2100F TEM. In addition, zeta potentials and hydrodynamic diameters of various samples were detected using a Zetasizer Nano ZS (Malvern). UV–visible absorption spectrum was measured using shimadzu UV-2600 UV–vis spectrophotometer. Fourier transform infrared spectroscopy (FT-IR) spectra were recorded on a Nicolet 7000-C spectrometer with KBr pellets. The powder X-ray diffraction (XRD) patterns were acquired on a PANalytical X’Pert PRO X-ray diffractometer, while X-ray photoelectron spectroscopy (XPS) measurements were conducted on a PHI 5000 VersaProbe spectrometer using a monochromatic Al Kα radiation source. In order to detect the photothermal effect, different concentrations of HMON@CuS, HMON@Gd or HMON@CuS/Gd in PBS solutions (200 μL) were irradiated with an 808 nm NIR laser (0.8 W/cm^2^, 5 min). The real-time temperatures of the solutions were recorded using a FLIR E50 camera system.

### Calculation of Photo-thermal Conversion Efficiency of HMON@CuS/Gd at 808 nm

The photothermal conversion efficiency of HMON@CuS/Gd was calculated by the following equation:

$$\eta = \frac{{hS(T_{ \hbox{max} } - T_{\hbox{max} ,water} )}}{{I(1 - 10^{{ - A_{808} }} )}} \times 100\%$$

where h is the heat transfer coefficient, S is the surface area of the container, T_max_ is the maximum equilibrium temperature for HMON@CuS/Gd NPs solution, while T_max,water_ is the maximum equilibrium temperature for water. In addition, I refer to the laser power (0.8 W/cm^2^), and A_808_ is the absorbance of the HMON@CuS/Gd NPs at 808 nm.

$$hS = \frac{{m_{D} \times C_{D} }}{{\tau_{S} }}$$

where m_D_ and C_D_ refers to the weight of water and the heat capacity of water respectively. In addition, τ_S_ is time constant of the sample.

$$t = - \tau_{S} \times { \ln }\theta ,{\kern 1pt} \quad \theta = \frac{{T_{amb} - T}}{{T_{amb} - T_{ \hbox{max} } }}$$

where T_amb_ is surrounding ambient temperature. Therefore, the τ_S_ can be calculated using the linear regression curve between cooling stage and negative natural logarithm of driving force temperature of HMON@CuS/Gd.

### Detection of singlet oxygen on solutions

After treated with or without NIR laser irradiation, the Singlet Oxygen Sensor Green reagent (Life Technologies, Carlsbad, CA, USA) was used to detect the production of singlet oxygen on the solutions contained HMON@CuS or HMON@CuS/Gd using a Jasco (Easton, MD, USA) FP-6200 spectrofluorometer.

### Biocompatibilities of HMON@CuS/Gd

To address biocompatibility issues, normal GES-1, BEAS-2B, LO2 and spleen cells were treated with PEG-modified or non-PEG-modified HMON@CuS/Gd solutions for 24 h. Then, cell viability was quantified with Cell Counting Kit-8 (CCK-8) assay (Dojindo, Tokyo, Japan) and a microplate reader. In order to evaluate immunotoxicity, the mouse macrophage (RAW264.7) cells were seeded into confocal laser scanning microscope (CLSM) dishes and treated with HMON@CuS/Gd for 24 h. Subsequently, treated cells were washed, fixed with 4% paraformaldehyde, treated with 1% bovine serum albumin (BSA), incubated with 0.1% Triton X-100(Abcam, Cambridge, UK), and finally stained with rhodamine-phalloidin (Abcam) and with 4′,6-diamidino-2-phenylindole (DAPI; Abcam). Finally, CLSM (Olympus, Tokyo, Japan) was used to observe and photograph stain cells.

### Cell uptake of HMON@CuS/Gd

In order to access the intracellular uptake efficiency, HGC-27 cells were planted in CLSM dishes, and then treated with fluorescein isothiocyanate (FITC)-labeled HMON@CuS/Gd, which were constructed as the followings: Briefly, 20 mg of HMON@CuS/Gd products were suspended with 20 mL ddH_2_O, and then 1 mg FITC were added and kept stirring for 8 h. After concentration and washing with ddH_2_O, the FITC-labeled HMON@CuS/Gd were constructed. After incubation for 1, 2 and 4 h, the cells were washed with PBS and collected. Following that, cells were directly analyzed by flow cytometry assay. For immunofluorescence imaging, treated cells were fixed with 4% paraformaldehyde, and stained with DAPI. Finally, The CLSM dishes were observed and imaged with CLSM (Olympus).

### In vitro photo-therapeutic effect of HMON@CuS/Gd

HGC-27 cells were pretreated with HMON@CuS/Gd, with or without an 808 nm laser at a power density of 0.8 W/cm^2^ for 5 min respectively. 24 h later, the treated cells were collected and incubated with CCK-8 kit for viabilities detection, with Annexin V-FITC/PI Kit (KeyGEN, Nanjing, China) for apoptosis detection and EDU testing kit (Ruibo, Guangzhou, China) for cell proliferation ability detection. Furthermore, in order to evaluate mitochondrial function, the treated cells were also harvested and detected with JC-1 Kit (Keygen), mitoROS kit (AAT Bioquest, Wuhan, China), ROS Kit (Keygen), mPTP kit (BestBio, Shanghai, China) follow the manual instructions.

### In vivo multi-modal imaging behaviors of HMON@CuS/Gd

In order to evaluate the multi-modal imaging behaviors of HMON@CuS/Gd, HGC-27 cells were harvested and suspended with appropriate amount of PBS, and then 50 μL of suspension was injected into the right flank of mice to construct tumor-bearing nude mice, which were randomly divided into different groups. For fluorescence images, the tumor-bearing mice were injected with DIR labeled HMON@CuS/Gd via tail vein injections, while DIR-labeled HMON@CuS/Gd were firstly constructed using similar methods of FITC labeled HMON@CuS/Gd. At the time points of 0, 3, 6, 12, 18 and 24 h after injection, FL images of whole body and their major organs were all detected by FL imaging system (Digital Fprcision Medicine Company, Beijing, China). For IRT images, the tumor-bearing mice were anesthetized before and after injecting with HMON@CuS/Gd, then thermographic images were captured by a FLIR E50 camera at the present of NIR laser irradiation. For MRI images, single HMON@CuS/Gd solutions, and tumor-bearing mice were anesthetized before and after injecting with HMON@CuS/Gd, then MRI images were captured through a 3.0 T MAGNETOM Skyra MRI scanner (Phihips, Amsterdam, Netherlands) using T1-weighted sequence (TR = 450.0 ms, TE = 15.3 ms, thickness = 2 mm).

All animal procedures were approved by Nanfang Hospital of Southern Medical University (Certification No. L2017243). All procedures of the investigation were carried out following the rules of the Declaration of Helsinki of 2008 (https://www.wma.net/what-we-do/medical-ethics/declarationofhelsinki/), revised in 2008.

### In vivo photo-therapeutic effect of HMON@CuS/Gd

In order to clarify the photo-therapeutic effect of HMON@CuS/Gd, tumor-bearing mice were randomly divided into four groups, which were treated with saline and HMON@CuS/Gd, with or without irradiation of NIR (0.8 W/cm^2^, 8 min). The body weight and tumor volume were recorded every 3 days for 14 days. At the 14th day, the mice were euthanized, then their tumor tissues were excised and fixed in 4% formalin for immunohistochemistry (IHC; Tunel, Abcam, USA) and H&E staining.

### Statistical analysis

Data are showed as mean ± standard deviation (SD). Quantitative analysis of the immunofluorescence intensity, western-blotting bands and IHC stained area by aniline was conducted using the Image J program (National Institutes of Health, Bethesda, MD). The differences between the mean values were analyzed using SPSS 21.0 (International Business Machines Corporation, Armonk, NY) and one-way ANOVO statistical approach. Results were considered statistically significant when *P *< 0.05. All experiments were repeated at least 3 times.

## Results and discussion

### Preparation, characterization and in vitro photo-thermal effect of HMON@CuS/Gd

**Fig. 1:**
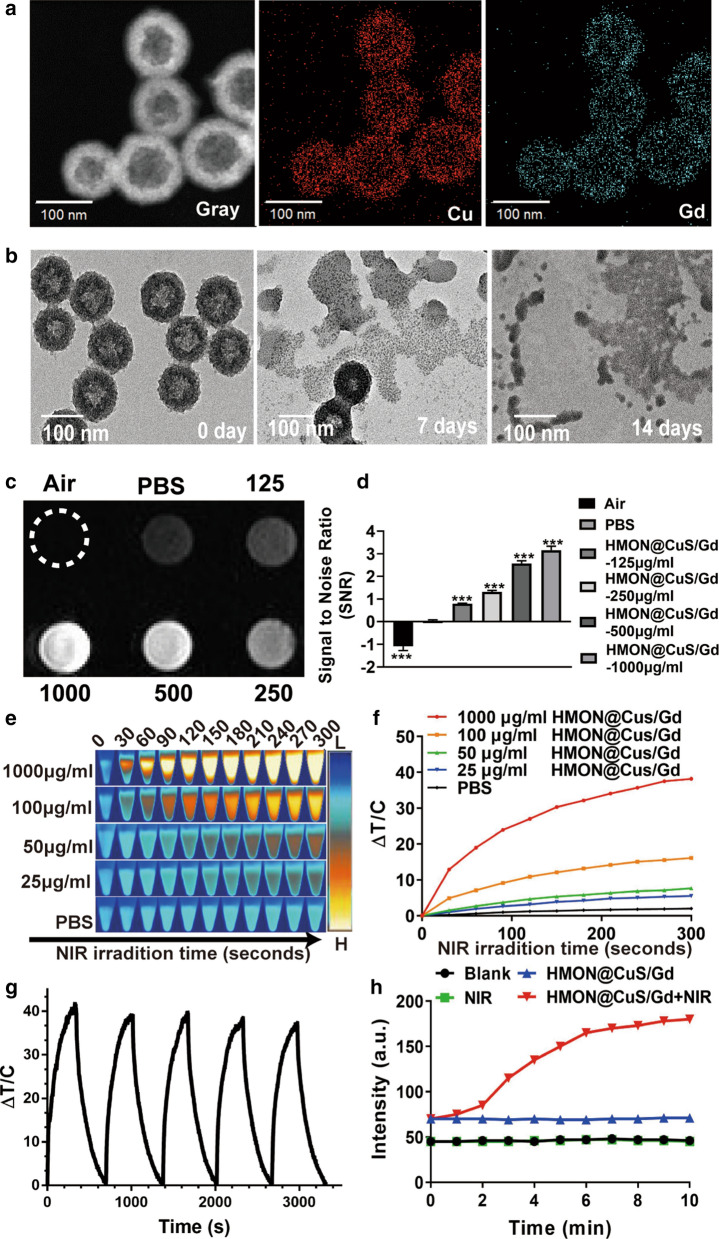
**a** Elemental mappings of Cu and Gd elements for HMON@CuS/Gd NPs. **b** TEM images of biodegradable HMON@CuS/Gd NPs immersed in 10 mM GSH aqueous solution for 7 days and 14 days. **c**, **d** T1-weighted MRI images of HMON@CuS/Gd at various concentrations (left) and the relative MR signal intensities (right). **e**, **f** The temperature increase curve induced by different concentrations of HMON@CuS/Gd aqueous solutions and PBS under NIR irradiation (0.8 W/cm^2^, 5 min). **g** PTT stability of HMON@CuS/Gd NPs under NIR laser irradiation (0.8 W/cm^2^, 5 min). **h** The production of singlet oxygen by the HMON@CuS/Gd NPs with or without NIR irradiation (0.8 W/cm^2^, 5 min). Data are shown as mean ± SD, n = 3

HMON were synthesized via a mild selective etching progress. In brief, mesoporous organosilica nanospheres with large pore were firstly synthesized by the dual hydrolysis and condensation of BTES and TEOS under the catalyzation of TEA, while CTAC was used for pore forming. As the dominant -Si–O- bonds were weaker than the –Si–C- bonds within the outer sites, the inner part of the mesoporous organosilica nanospheres were then etched using diluted ammonia to form the hollow mesoporous structure, bridged by –S–S– bonds [21]. Though it has been reported that CuS could be conjugated onto the surface of HMON through thiol groups, it required respective synthesis of HMON and CuS following the conjugation progress. The multi-steps protocol might be simplified as it has also been reported that Si–O– groups on the surface of the HMON have the potential to bond with metal ions (such as Fe^3+^, Au^+^, etc.) without the need of additional molecular chelator [27, 28]. In our previous work, CuS nanocrystals were in situ grown onto the surface of hollow mesoporous TaOx nanospheres without the help of thiol groups [19]. Herein, CuS nanocrystals were in situ grown onto the surface of the HMON similarly. Briefly, Cu^2+^ ions were added directly into the HMON solution and adsorbed onto the HMON, following the addition of Na_2_S to achieve the in situ nucleation and growth of CuS nanocrystals. Finally, the HMON@CuS/Gd NPs were constructed with the addition of GdCl_3_·6H_2_O. Then, the element mapping of HMON@CuS/Gd showed that the Cu^2+^ and Gd^3+^ ions were successfully jointed together onto the surface of HMON (Fig. 1a). It is noticeable that the etching degree by diluted ammonia has great influence on the ability of the HMON to absorb Cu^2+^ ions. HMON with excessive etching leading to the poor conjugation with Cu^2+^ and Gd^3+^ ions due to the depletion of –Si–O– bonds. Eventually, C18PMH-mPEG was applied to improve the stability and dispersity of the HMON@CuS/Gd nanospheres.

The as-prepared HMON showed specific strong and broad peaks at 2927.32 cm^−1^ and 2855.28 cm^−1^ (attributed to –CH_2_) [29], which represented the covalent grafting of C18PMH-mPEG chains onto the surface of HMON, compared to non-PEGylation HMON. Meanwhile, new peak at 627.03 cm^−1^ (attributed to Cu–S) was also observed in HMON@CuS and HMON@CuS/Gd [30]. Therefore, the FI-TR results consistently confirmed the successful synthesis of HMON@CuS/Gd (Additional file 1: Figure S1). UV–vis spectra results revealed that HMON@CuS and HMON@CuS/Gd both have strong absorption in NIR region (Additional file 1: Figure S2), which is mainly contributed to the effective adherence of CuS nanocrystals on the surface of HMON [21]. XPS results demonstrated the presence of C (C 1 s peaks at 284.95 eV), O (O 1 s peaks at 531.99 eV), Si (Si 2p peaks at 102.53 eV), Cu (Cu 2p peaks at 933.27 eV), S (S 2p peaks at 163.36 eV) and Gd (Gd 4d peaks at 142.72 eV) (Additional file 1: Figure S3) [31]. From XRD results, HMON@CuS and HMON@CuS/Gd both showed specific characteristic diffraction peaks [30], which is indexed as a typical covellite crystalline phase of CuS (Additional file 1: Figure S4). It is noteworthy that CuS crystal was found in HMON@CuS and HMON@CuS/Gd, while no Gd-related crystal was observed in HMON@CuS/Gd, indicating that Gd was doped in HMON, but not as a crystal form. Taken together, the above results consistently confirmed the successful synthesis of HMON@CuS/Gd. In addition, the diameters of HMON was 115.5 ± 1.46 nm, while CuS or/and Gd^3+^ loading led to a slight increase in the size of the HMON@CuS (116.7 ± 1.51 nm) and HMON@CuS/Gd (117.1 ± 1.71 nm), and all of them exhibited unimodal size distribution. Meanwhile, the data also indicated that both of HMON@CuS and HMON@CuS/Gd possessed negative net charge, which could help to improve their circulation stability (Additional file 1: Figure S5). Compared to these PEG modified HMON NPs, HMON-based materials without PEG modification also showed negative net charge, and similar diameters. However, the diameters’ SD value in HMON (108.2 ± 4.54 nm), HMON@CuS (109.4 ± 3.41 nm) and HMON@CuS/Gd (110.1 ± 4.15 nm) NPs were larger than those in PEG-modified HMON NPs, indicating that C18PMH-mPEG could improve their stability (Additional file 1: Figure S6). Consequently, PEG modified HMON NPs were chosen for further experiments in our research. Due to the presence of the disulfide bonds in the framework of HMON, HMON@CuS/Gd exhibited time-dependent biodegradable behavior in the glutathione (GSH) solutions (Fig. 1b), indicating that HMON@CuS/Gd NPs could be bio-degradable and eliminated through feces and urine, thus the drug accumulation dependent toxicity could be avoided.

It could also be expected that the as-prepared NPs possess the potential to be applied as MRI imaging contrast agent due to the addition of Gd [32, 33]. In vitro experiment demonstrated that T1 signal intensity was enhanced with the increasing concentration of HMON@CuS/Gd solutions. As calculated, significant increase of SNR (signal to noise ratio) could also be observed, indicating the outstanding potential for MRI imaging (Fig. 1c, d). Besides the MRI imaging properties, we further investigated the photo-thermal conversion efficiency of HMON@CuS/Gd by monitoring the temperature changes under NIR laser irradiation in vitro using an infrared thermal imaging camera. After NIR laser irradiation (0.8 W/cm^2^) for 5 min, dramatical temperature increase was observed in HMON@CuS/Gd and HMON@CuS groups, while no obvious temperature change was shown in PBS or HMON@Gd groups with the same laser irradiation. The maximum increased temperature (ΔT_max_) of HMON@CuS/Gd and HMON@CuS groups (1000 µg/mL) ∼ 38 °C, whereas the ΔT_max_ of other HMON@CuS/Gd and HMON@CuS groups (100 µg/mL, 50 µg/mL and 12.5 µg/mL) increased to ∼ 16.0 °C, ∼ 8.0 °C and ∼ 6.0 °C respectively (Fig. 1e, f; Additional file 1: Figure S7). It is worth pointing that HMON@CuS/Gd and HMON@CuS showed good temperature response under the NIR laser irradiation conditions, which demonstrating that the photo-thermal conversion efficiency was contributed to the presence of CuS. Moreover, HMON@CuS/Gd NPs showed an excellently stable photo-thermal performance, as the heat-cool curve of HMON@CuS/Gd NPs had no significant difference within five cycles of NIR laser irradiation (Fig. 1g). According to the linear regression curve between the cooling stage and negative natural logarithm of driving force temperature of HMON@CuS/Gd, the photo-thermal conversion efficiency of HMON@CuS/Gd NPs was calculated to be 82.4% (Additional file 1: Figure S8). In addition, to determine whether HMON@CuS/Gd NPs could produce ROS under NIR laser irradiation, a singlet oxygen sensor was applied to detect ROS levels. As shown in Fig. 1h, HMON@CuS/Gd NPs exhibited concentration-dependent production of ROS in deionized water, indicating that HMON@CuS/Gd NPs possessed excellent PDT ability. Moreover, HMON@CuS showed similar ROS generation ability in the presence of NIR, while no excessive singlet oxygen was observed in HMON@Gd NPs, with or without NIR treatment (Additional file 1: Figure S9), which demonstrating that the ROS generation capacity of HMON@CuS/Gd NPs were contributed to the presence of CuS. Therefore, HMON@CuS/Gd would be promising PTT, PDT and functional imaging NPs for the treatment of GC.

### In vitro photo-therapeutic effect of HMON@CuS/Gd nanoparticles in GC cells

**Fig. 2:**
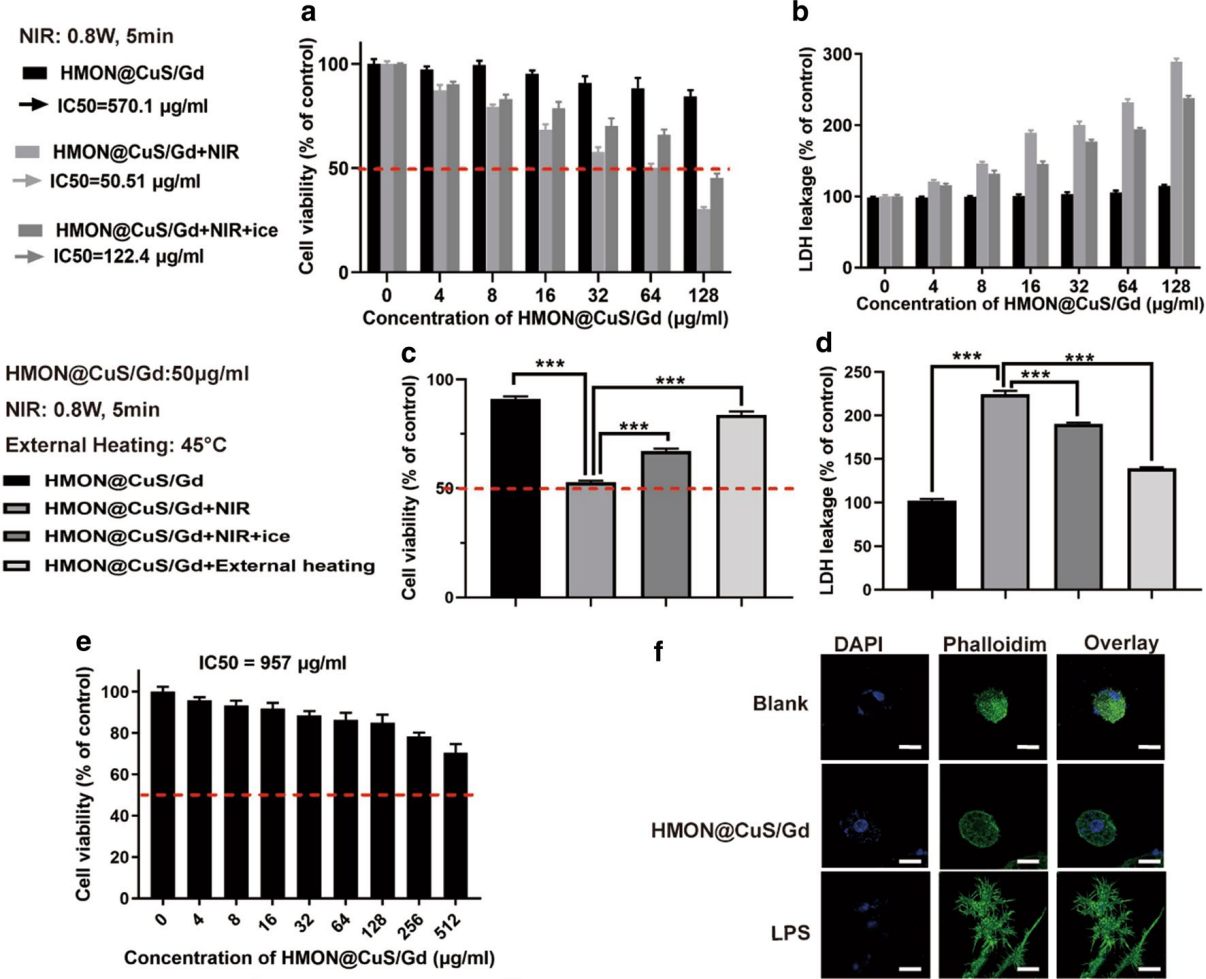
**a** Cell viabilities of HGC-27 cells after treated with single HMON@CuS/Gd, HMON@CuS/Gd plus NIR and HMON@CuS/Gd plus NIR on ice, using CCK-8 assay. **b** LDH leakage of HGC-27 cells after treated with single HMON@CuS/Gd, HMON@CuS/Gd plus NIR and HMON@CuS/Gd plus NIR on ice, using LDH assay. **c** CCK-8 assay of HGC-27 cells after treated with single HMON@CuS/Gd, HMON@CuS/Gd plus NIR, HMON@CuS/Gd plus NIR on ice or HMON@CuS/Gd plus external heating. **d** LDH leakage assay of HGC-27 cells after treated with single HMON@CuS/Gd, HMON@CuS/Gd plus NIR, HMON@CuS/Gd plus NIR on ice or HMON@CuS/Gd plus external heating. **e** The CCK-8 assay of GES-1 cells after incubation with HMON@CuS/Gd NPs for 24 h. **f** The confocal laser scanning microscopy (CLSM) images of RAW264.7 murine macrophage-like cells after incubation with HMON@CuS/Gd for 24 h. Scale bar: 20 μm. Data are shown as mean ± SD, n = 3. * indicates *P *< 0.05, *** indicates *P *< 0.001

Firstly, we tested the in vitro photo-therapeutic effects of HMON@CuS/Gd on HGC-27 cells. Briefly, HGC-27 cells were first incubated with HMON@CuS/Gd at different concentrations for 4 h and then were irradiated with an 808 nm laser at a power density of 0.8 W/cm^2^ for 5 min. After 24 h, CCK-8 assay was applied to evaluate the antitumor therapeutic efficacies of HMON@CuS/Gd. As shown in Fig. 2a, free HMON@CuS/Gd and single NIR laser treatment did not induce significant changes in cell death, since the half maximal inhibitory concentration (IC50) of HMON@CuS/Gd was as high as 570.01 µg/mL. Furthermore, dramatical decreased cell viabilities were observed in HMON@CuS/Gd plus NIR group (IC50 = 50.51 µg/mL), indicating that HMON@CuS/Gd exhibited an effective photo-therapy effect with NIR irradiation. In the other hand, HMON@CuS exhibited similar anti-tumor effect with NIR irradiation, compared to HMON@CuS/Gd plus NIR irradiation group, indicating that photo-therapy effect was contributed to the addition of CuS, while Gd showed negligible cell toxicity (Additional file 1: Figure S10). Consistently, LDH assay revealed that HMON@CuS/Gd plus NIR induced higher levels of LDH leakage than blank group, while HMON@CuS/Gd only and single NIR laser did not induce any LDH changes (Fig. 2b). These results suggested that HMON@CuS/Gd could provide a promising killing effect to GC cells, with the irradiation of NIR.

As it has been reported, several CuS-based NPs had been applied for PDT&PTT synergistic therapy [34, 35]. However, the therapeutic effect of HMON@CuS-induced PTT and PDT at mild temperature, has not been fully discussed. It is still wondered whether the therapeutic effect was achieved by PTT ablation alone, or PDT also played some certain role in the therapeutic progress. In order to evaluate the PDT effect, HGC-27 cells were placed on an ice box while being irradiated with NIR light and the temperature was controlled below 10 °C to minimize the effect of NIR-induced PTT ablation. To our surprise, significant cell death was still observed in this condition, though not as obvious as HMON@CuS/Gd plus NIR treatment, as IC50 raised to 122.4 µg/mL (Fig. 2c). Furthermore, evident LDH leakage was also found, shown in Fig. 2d.

**Fig. 3:**
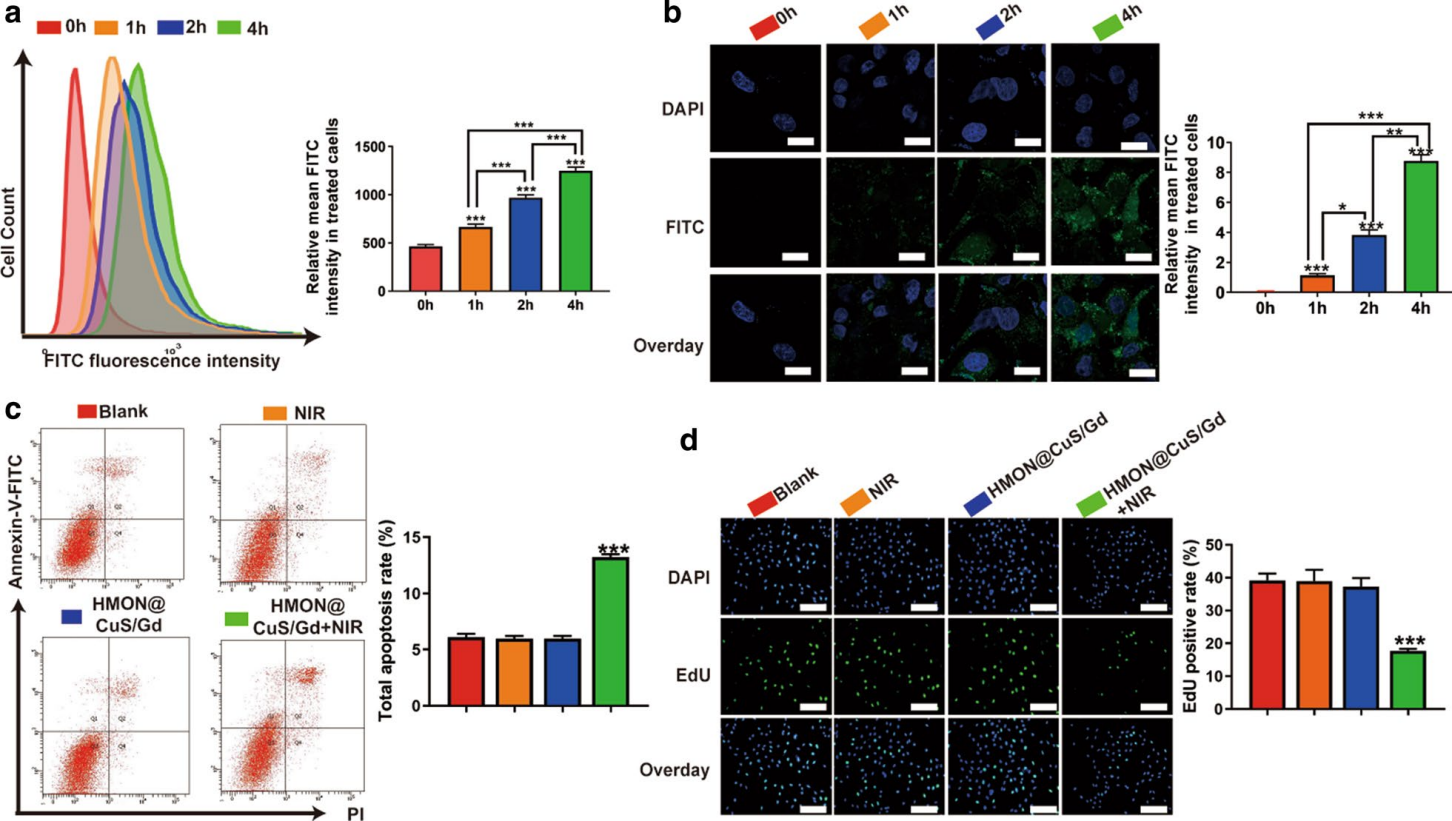
**a** Flow cytometry images of HGC-27 cells treated with HMON@CuS/Gd for 1–4 h (left), and corresponding quantification of green mean fluorescence intensity of FITC inside cells (right). **b** CLSM images of HGC-27 cells treated with HMON@CuS/Gd for 1–4 h (left), and corresponding quantification of CLSM images using the green mean fluorescence intensity of FITC (right). Scale bar: 30 μm. **c** Cell apoptosis images of HGC-27 cells (left) and statistical graph of total apoptotic rates (right), after treated with or without near infrared (NIR) irradiation, in the presence of HMON@CuS/Gd nanoparticle. **d** EdU images of HGC-27 cells (left) and statistical graph of EdU-positive rates (right), after treated with or without near infrared (NIR) irradiation, in the presence of HMON@CuS/Gd nanoparticle. Scale bar: 100 μm. Data are shown as mean ± SD, n = 3. *** indicates *P *< 0.001

When referring to PTT effect, clinicians have pointed out that tumor cells might occur apoptosis under 43–45 °C, while normal cells are generally tolerant to this temperature condition [36]. In another word, PTT effect refers to generating external 45 °C heating at cancer region, which is supposed to have a direct cytotoxic effect on tumor cells, and enhance the efficacy of chemotherapy and radiotherapy, improve the body’s immunity, and thus suppress tumor progression [18, 37]. Coincidentally, it could be observed by the IR camera that when HGC-27 cells were treated with HMON@CuS/Gd (50 µg/mL) plus NIR irradiation (0.8 W/cm^2^, 5 min), the temperature could raise up to around 45 °C at 37 °C temperature condition. Consequently, HMON@CuS/Gd with 50 µg/mL concentration were chosen for the following cellular experiments. Simulated 45 °C external temperature condition using a cell culture incubator was set as control. It could be seen that slight cell death and LDH leakage were observed in the external heating group, comparing with NIR irradiation group, indicating hyperthermia might not be the only factor (Fig. 3c, d). Taken together, it could be concluded that HMON@CuS/Gd could induce combined PDT and PTT effect, while this obvious anti-tumor cell death was contributed to their PDT&PTT synergetic effect.

To address biocompatibility issues, in vitro experiments were performed by incubating HMON@CuS/Gd NPs with normal GES-1 cells. CCK-8 assay revealed above 85% cell viability rates for GES-1 as the concentration ranging from 0 to 512 µg/mL, while the IC50 of HMON@CuS/Gd was as high as 957 µg/mL to GES-1 cells (Fig. 2e). However, when treated with non-PEG modified HMON@CuS/Gd NPs, cell viability rates for GES-1 cells showed significant decrease (IC50 = 312 µg/mL; Additional file 1: Figure S11), indicating the addition of PEG could improve their bio-safety, and PEG modified HMON@CuS/Gd were consequently chosen for further biological experiments in our research. Surprisingly it could be observed that IC50 to GES-1 cells was much higher than HGC-27 GC cells, indicating that the as-prepared HMON@CuS/Gd showed specific toxicity to GC cell. Furthermore, HMON@CuS/Gd (50 µg/mL) were also co-incubated with RAW264.7 murine macrophage-like cells. This immunotoxicity experiment revealed that HMON@CuS/Gd treatment did not elicit any inflammatory response at the cellular level (Fig. 2F). These results strongly indicated that HMON@CuS/Gd NPs have good in vitro biocompatibilities, highlighting their value for clinical translation as drug carriers.

### In vitro cellular uptake and anti-GC effect of HMON@CuS/Gd nanoparticles

Cellular uptake of HMON@CuS/Gd were then investigated by flow cytometry and CLSM assays, while FITC were firstly encapsulated into HMON@CuS/Gd NPs. After incubating with HMON@CuS/Gd (50 µg/mL) for 1–4 h, the FITC fluorescence intensities increased significantly as time passed by (Fig. 3a, b), indicating the excellent cellular uptake efficiency of HMON@CuS/Gd. Since HMON@CuS/Gd could efficiently enter GC cells, we further detected the apoptosis rates determined by flow cytometry, which demonstrated that HMON@CuS/Gd plus NIR laser irradiation induced twofold higher levels of total apoptosis (14%) than blank group (5%), while free HMON@CuS/Gd and single NIR laser did not induce any apoptotic change (Fig. 3c). EdU dye is a kind of thymidine nucleoside analogues, which could specifically insert into DNA molecules of rapid proliferation cells, and higher EdU-positive cell rates usually demonstrate better cell growth abilities [38]. In our research, after conjugated reactions with EdU, we found that HMON@CuS/Gd plus NIR laser irradiation induced twofold lower levels of EdU-positive rates (15%) than blank group (40%), while free HMON@CuS/Gd NPs and single NIR laser did not induce any EdU-positive rates change (Fig. 3d). These results suggested that HMON@CuS/Gd provided a promising anti-proliferation and promoting apoptotic effects to GC cells, with the irradiation of NIR.

### Anti-tumor mechanism of photo-therapeutic effect induced by HMON@CuS/Gd

**Fig. 4:**
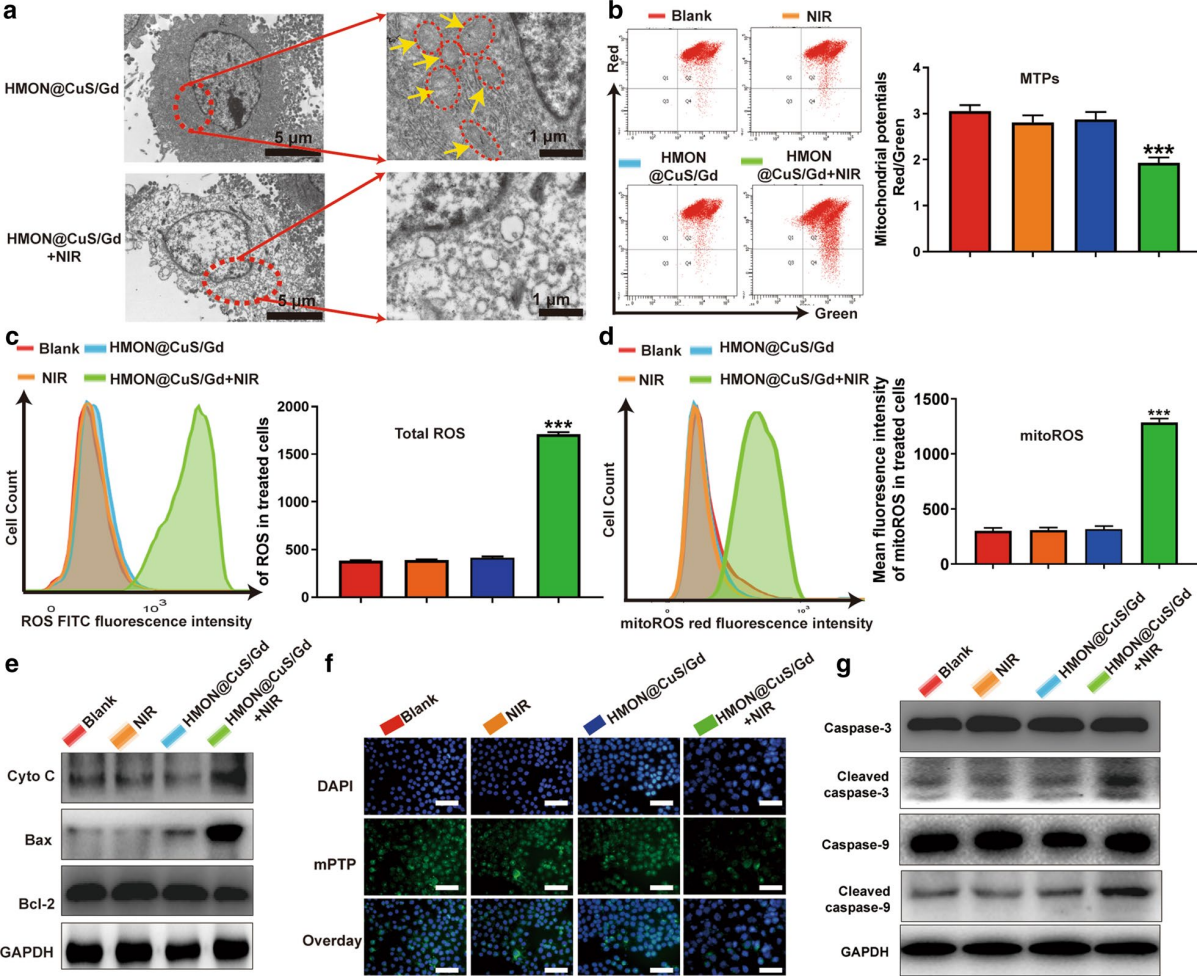
HGC-27 cells were treated with or without NIR irradiation, in the presence of HMON@CuS/Gd nanoparticle, while divided into four groups, including blank, HMON@CuS/Gd, sing NIR and HMON@CuS/Gd plus NIR treatments. **a** TEM images of HGC-27 cells. **b** MTPs (left) and statistical graph of total MTPs (right) in HGC-27 cells, detected by flow cytometry. **c** Total ROS images of HGC-27 cells (left), and statistical graph of ROS content in HGC-27 cells (right), detected by flow cytometry. **d** MitoROS images of HGC-27 cells (left), and statistical graph of mitoROS content in HGC-27 cells (right), detected by flow cytometry. **e** Expressions of Cyto C, Bax, Bcl-2 and GAPDH proteins in HGC-27 cells, detected by western blotting. **f** MPTP images of HGC-27 cells, detected by fluorescent inverted microscope. Scale bar: 100 μm. **g** Expressions of caspase-9, cleaved caspase-9, caspase-3, cleaved caspase-3, and GAPDH proteins in HGC-27 cells, detected by western blotting. Data are shown as mean ± SD, n = 3. *** indicates *P *< 0.001

Though excellent anti-tumor effect has been proved, their relevant photo-therapeutic mechanism remained unknown. To examine the cancer-killing mechanism of HMON@CuS/Gd, we incubated HGC-27 cells with HMON@CuS/Gd (50 µg/mL) for 4 h, irradiated with an 808 nm laser (0.8 W/cm^2^, 5 min), then observed by TEM. Surprisingly, dozens of mitochondria were shown in non-treated HGC-27 cells, while no mitochondria were observed in treated HGC-27 cells (Fig. 4a). Meanwhile, it could be proved that MTPs of HGC-27 cells was reduced by HMON@CuS/Gd NPs with the assistance of NIR irradiation, further indicating the damage of mitochondria (Fig. 4b). Consequently, we assumed that HMON@CuS/Gd induced PTT&PDT probably resulted in mitochondrial damage to exert its function.

Mitochondria are central organelles for the regulation of cancer cell life and death, to which the damage can directly activate the intrinsic apoptosis pathway. When cells receive certain external stimuli, the mitochondrial electron transport is blocked, MTPs changes, the maintain of mPTP is disrupted and Cyto c release, followed by activates caspase-depended apoptosis pathway [39]. Actually, the mitochondrial-dependent damage could be triggered via a range of exogenous and endogenous stimuli, such as oxidative stress, ischemia and DNA damage [40, 41]. As it is well-known, ROS are inevitable products of cell metabolism, while high levels of intracellular ROS could attack mitochondria and cause mitochondrial-damage-dependent apoptosis [42]. Accumulating studies had proved that NIR-mediated PTT&PDT could induce the outbreak of ROS, following by activating oxidative stress and mediating mitochondrial damage in cancer cells [15, 16, 43]. As it has been demonstrated in the CCK-8 results, HMON@CuS/Gd did induce obvious anti-tumor cell death, partly contributed by their PDT effect. In order to clarify the ROS generation, DCFH-DA was applied as biological probe to monitor the intracellular level of ROS [44]. Increasing ROS generation was found to be significantly increased in HMON@CuS/Gd plus NIR treated HGC-27 cells (Fig. 4c). To further identify the PDT effect, HGC-27 cells were placed on an ice box while being irradiated with NIR light, and the temperature was controlled below 10 °C to minimize the effect of NIR-induced PTT ablation. Interestingly, dramatical increase of ROS was still observed in this condition, though not as obvious as HMON@CuS/Gd plus NIR treatment. Meanwhile, our results showed that slight ROS was produced in the external heating group (Additional file 1: Figure S12). Consequently, it could be concluded that HMON@CuS/Gd could induce ROS generation, was also mainly contributed to their PDT effect.

Meanwhile, in order to clarify whether the generated ROS could cause mitochondrial dysfunction, MitoSOX Red was used as an indicator to discern the superoxide in mitochondria through flow cytometry analysis (FACS) in this study [45]. Notably, a sharp increase in red fluorescence (refer to mitochondrial ROS, also called mitoROS) was detected in HMON@CuS/Gd plus NIR group (Fig. 4d). Taken together, we assumed that photo-therapeutic effect induced by HMON@CuS/Gd NPs might promote intracellular ROS level and induce mitochondrial dysfunction to some extent. The formation of much more amount of mitoROS species was also discovered, which in turn aggravate the damage of mitochondria and might activate the Caspase-depended apoptosis pathway. To verify whether the Caspase-depended apoptosis pathway was activated, the expression of pro-apoptotic protein (Bax) and anti-apoptotic protein (Bcl-2) was detected. It could be seen that pro-apoptotic protein (Bax) dramatically elevated and anti-apoptotic protein (Bcl-2) decreased (Fig. 4e; Additional file 1: Figure S13). As Bax was a pro-apoptotic protein with multiple Bcl-2 homology domains, this alteration could alter mPTP [46] (Fig. 4f; Additional file 1: Figure S14). MPTP opening is the primary event of the mitochondrial intrinsic apoptosis pathway, could be referred as sudden mitochondrial permeability transition and loss of inner mitochondrial potential, leading to the increase of cytochrome c (Cyto C) (Fig. 4e; Additional file 1: Figure S13). Considering Cyto c release from mitochondria to the cytoplasm has been verified as the most important event in caspase depended mitochondrial mediated apoptosis signal transduction pathway, the expression of apoptotic proteins in treated cells were further detected. From the western blot results, the scale of the expressed well-defined apoptosis protein markers (cleaved caspase-9/caspase-9, cleaved caspase-3/caspase-3) was markedly increased in HMON@CuS/Gd plus NIR group, compared with other three groups (Fig. 4g; Additional file 1: Figure S15). Through the involvement of caspase-9, Caspase-3 is subsequently cleaved, which could activate DNA fragmentation, thereby inducing cell apoptosis [47, 48]. Taken together, our research demonstrates that HMON@CuS/Gd plus NIR treatment did induce mitochondrial damage to trigger GC cells’ caspase-dependent apoptosis pathway.

**Fig. 5:**
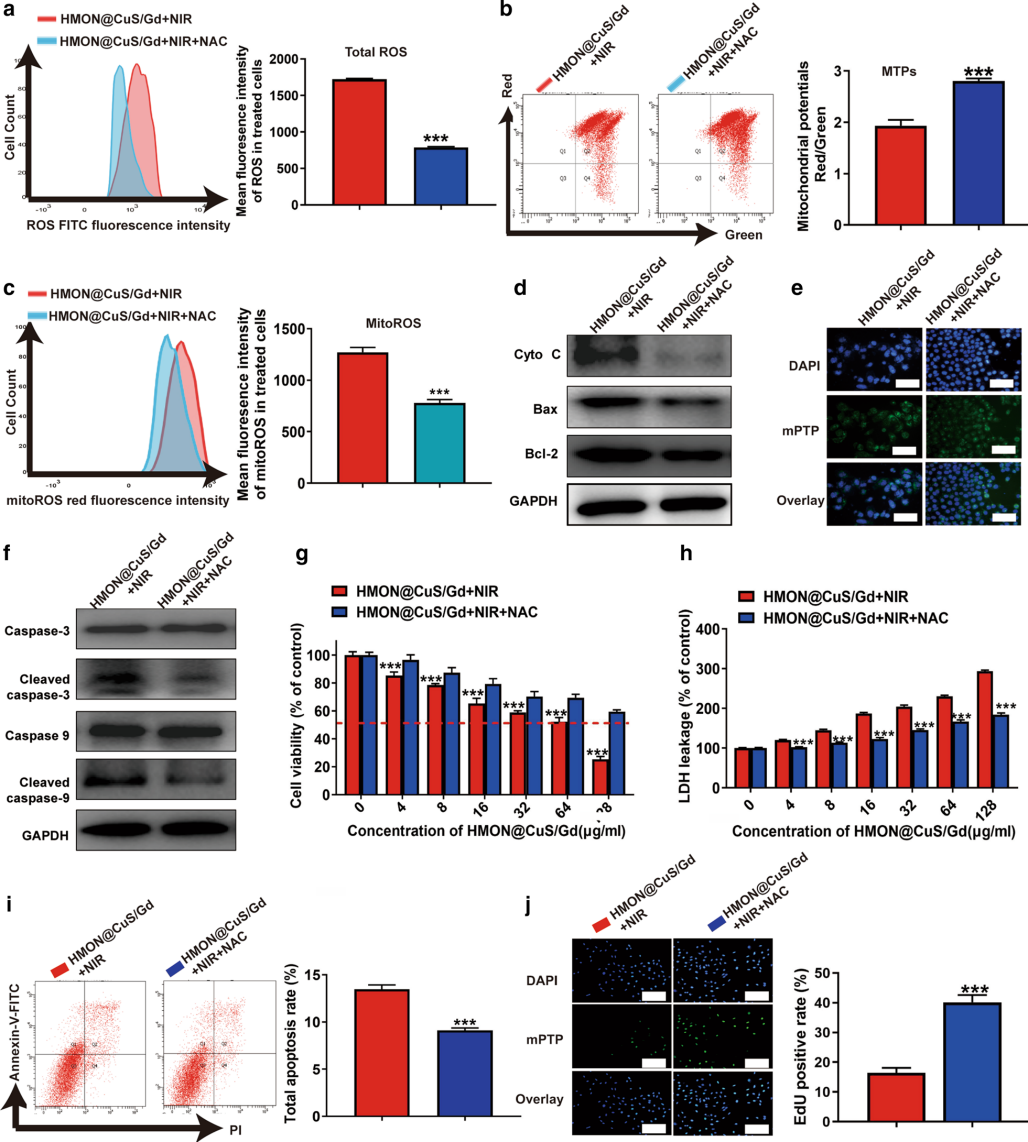
HGC-27 cells were treated with HMON@CuS/Gd plus NIR irradiation, with or without the addition of NAC. **a** Total ROS images of HGC-27 cells (left), and statistical graph of ROS content in HGC-27 cells (right), detected by flow cytometry. **b** MTPs images of HGC-27 cells (left), and statistical graph of MTPs in HGC-27 cells (right), detected by flow cytometry. **c** MitoROS images of HGC-27 cells (left), and statistical graph of mitoROS content in HGC-27 cells (right), detected by flow cytometry. **d** Expressions of Cyto C, Bax, Bcl-2 and GAPDH proteins in HGC-27 cells, detected by western blotting. **e** MPTP images of HGC-27 cells, detected by fluorescence microscope. Scale bar: 100 μm. **f** Expressions of caspase-9, cleaved caspase-9, caspase-3, cleaved caspase-3, and GAPDH in HGC-27 cells, detected by western blotting. **g** Cell viabilities of HGC-27 cells, detected by CCK-8 assay. **h** LDH leakage of HGC-27 cells, detected by LDH assay. after treated with or without NAC, in the presence of HMON@CuS/Gd plus NIR treatment. **i** Cell apoptosis images of HGC-27 cells (left) and statistical graph of total apoptotic rates (right), detected by flow cytometry. **j** EdU images of HGC-27 cells (left) and statistical graph of EdU-positive rates (right). Scale bar: 100 μm. Data are shown as mean ± SD, n = 3. *** indicates *P *< 0.001

In order to further confirm whether the mitochondrial-damage-dependent apoptosis pathway contributed by HMON@CuS/Gd induced PDT effect, *N*-Acetyl-l-cysteine (NAC, an anti-oxidant containing sulfhydryl group) was applied for the rescue experiments [49]. With the addition of NAC, intracellular ROS level in HMON@CuS/Gd plus NIR treated HGC-27 cells was dramatically decreased (Fig. 5a). Interestingly, MTPs, mitoROS and mPTP in treated HGC-27 cells was consistently reversed (Fig. 5b, c, e; Additional file 1: Figure S16). Furthermore, we also found the expression of apoptosis protein markers (Bax) and Cyto C was significantly decreased, while the anti-apoptotic protein (Bcl-2) increased (Fig. 5d; Additional file 1: Figure S17), which both indicated that mitochondrial damage was partly rescued. Since Cyto C has been reported to be one of the main activators during the caspase-dependent cell death, caspase-pathway would be inhibited if Cyto C’s release was decreased. As expected, the expression of apoptosis protein markers (cleaved caspase-9/caspase-9, cleaved caspase-3/caspase-3) were significantly decreased (Fig. 5f; Additional file 1: Figure S18). Furthermore, cell viabilities, LDH leakage, cell proliferation and apoptotic levels were consistently reversed, when ROS was suppressed by NAC (Fig. 5g–j). In all, these results indicated that HMON@CuS/Gd plus NIR could exert anti-proliferation effect through activating the ROS/mitochondria-damage/caspase pathway.

### In vivo multi-mode imaging behaviors of HMON@CuS/Gd nanoparticles

**Fig. 6:**
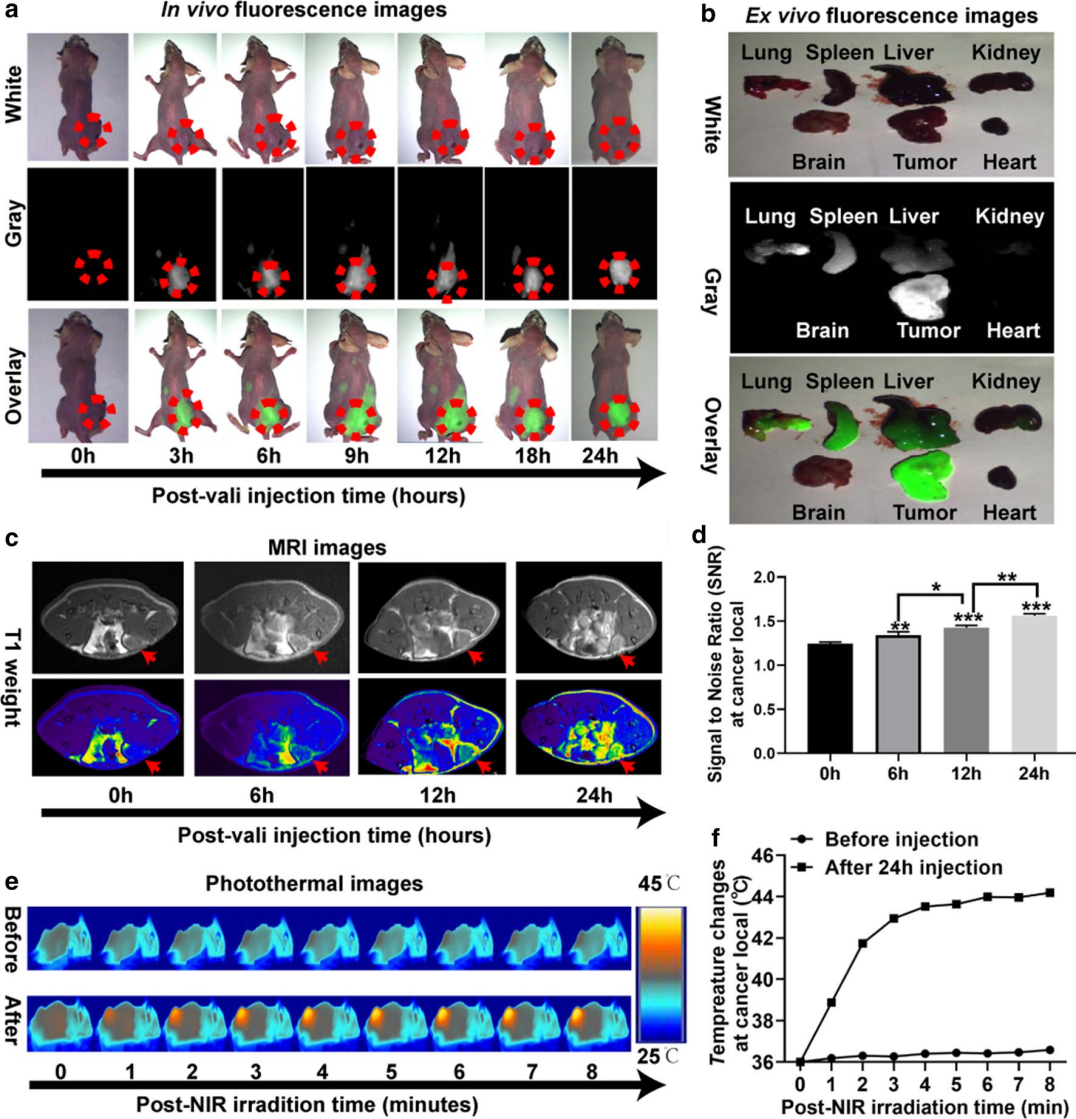
In vivo multi-mode imaging behaviors of HMON@CuS/Gd NPs HGC-27 cells. **a** In vivo fluorescence images of HGC-27 tumor bearing mice at 0, 3, 6, 12, 18 and 24 h after treatment with HMON@CuS/Gd. **b** Ex vivo Fluorescence image of major organs on mice. **c**, **d** In vivo MRI images of HGC-27 tumor bearing mice at 0, 6, 12 and 24 h after treatment with HMON@CuS/Gd, and the statistial signal to noise ratio (SNR) at cancer region. **c**, **d** In vivo MRI images of HGC-27 tumor bearing mice at 24 h after treatment with HMON@CuS/Gd (**c**; left), and the statistical signal to noise ratio (SNR) at cancer region (**d**; right). **e**, **f** In vivo IRT images of HMON@CuS/Gd-treated HGC-27 tumor bearing mice at different time, ranging from 0 to 8 min with NIR irradiation (**e**; left), and the statistical temperature change at cancer region (**f**; right)

Before moving forward to study in vivo tumor photo-therapy, in vivo biodistribution behaviors of such agent in HGC-27 tumor-bearing mice were studied. DIR, as a near-infrared lipophilic carbocyanine dye, has been used for many cell biology applications [50, 51]. DIR were first encapsulated into HMON@CuS/Gd NPs (HMON@CuS/Gd-DIR). Following that, tumor-bearing mice were treated with HMON@CuS/Gd-DIR via tail vein injections. After 0, 3, 6, 9, 12, 18 and 24 h, the mice were imaged using a small animal in vivo fluorescence imaging system (DIGITAL FPRCISION MEDICINE Company, Beijing, China). The in vivo fluorescence images indicate that HMON@CuS/Gd could access the cancer region in 3 h, then gradually enriched in the tumor site from 3 to 24 h (Fig. 6a; Additional file 1: Figure S19). As shown in Fig. 6a, a significant fluorescence signal accumulated in the tumor in the HMON@CuS/Gd-treated mice and peaked at 24 h, which provided an optimal therapeutic time window for subsequent therapy in vivo. Meanwhile, tumor-bearing mice were sacrificed 24 h post injection, and major organs were collected for fluorescence imaging. Ex vivo imaging also showed that HMON@CuS/Gd NPs were mainly accumulated in the tumor region (Fig. 6b), which further confirming that their passive tumor-targeting abilities through enhanced permeability and retention (ERP) effect [52, 53]. Since HMON@CuS/Gd NPs were accumulated in lung, spleen and liver, we have already carried out CCK-8 assays to further test the biocompatibility of HMON@CuS/Gd NPs, by incubating HMON@CuS/Gd with lung cells (BEAS-2B), primary spleen cells of mice (spleen cells) and liver cells (LO2). From the in vitro results, all the cells showed above 85% cell viabilities rates, which indeed confirm the biosafety of HMON@CuS/Gd NPs (Additional file 1: Figure S20). Moreover, to further demonstrate the enhancement of MRI, HGC-27 tumor-bearing mice were also detected using a 3.0 T MAGNETOM Skyra MRI scanner, as it has been verified that HMON@CuS/Gd could be used as MRI T1 contrast agent in vitro. HMON@CuS/Gd solution (6 mg kg^−1^) was tail-intravenous injected, and then MRI images of the tumor were obtained after 24 h. Figure 6c, d displayed that HMON@CuS/Gd could significantly enhanced T1-weighted signal after tail-intravenous administered, and these results not only highlighted the potential of HMON@CuS/Gd for MRI, but also again confirmed that HMON@CuS/Gd could gradually access the cancer region within 24 h, and 24 h could serve as an optimal therapeutic time window for subsequent therapy in vivo. Subsequently, the IRT imaging abilities of HMON@CuS/Gd in vivo were further investigated. HGC-27 tumors on mice that were exposed to NIR irradiation (0.8 W/cm^2^, 8 min), after tail-intravenous injected with HMON@CuS/Gd for 24 h. As shown in Fig. 6e, f, the tumor temperature of tumors increased from ∼ 36 to ∼ 44 °C within 3 min, while maintained at 43–45 °C in the next 5 min. This temperature was much greater than that (36 °C) experienced by mice administered saline plus NIR irradiation (0.8 W/cm^2^, 8 min), indicating that HMON@CuS/Gd did exhibit mild PTT effect (43–45 °C) under NIR irradiation (under 0.8 W/cm^2^), which would be much more attractive for clinical photo-therapeutic treatment.

Development of multifunctional nanoplatforms integrating both diagnostics and treatment functions for cancer nano-theranostics have attracted widespread research interest in nano-biotechnology [54, 55]. However, it is still worth to construct theranostic nano-systems with multi-modal imaging to guide therapy. Our results revealed that HMON@CuS/Gd did exhibit good FL, MRI and IRT imaging capacities. Furthermore, HMON@CuS/Gd NPs could selectively cause PT at cancer region, guided by FL/MRI/IRT imaging. Taken together, HMON@CuS/Gd NPs might be a promising treatment for solid tumors with precise photo-therapeutic efficiency.

### In vivo photo-therapeutic treatments of HMON@CuS/Gd nanoparticles in GC

**Fig. 7:**
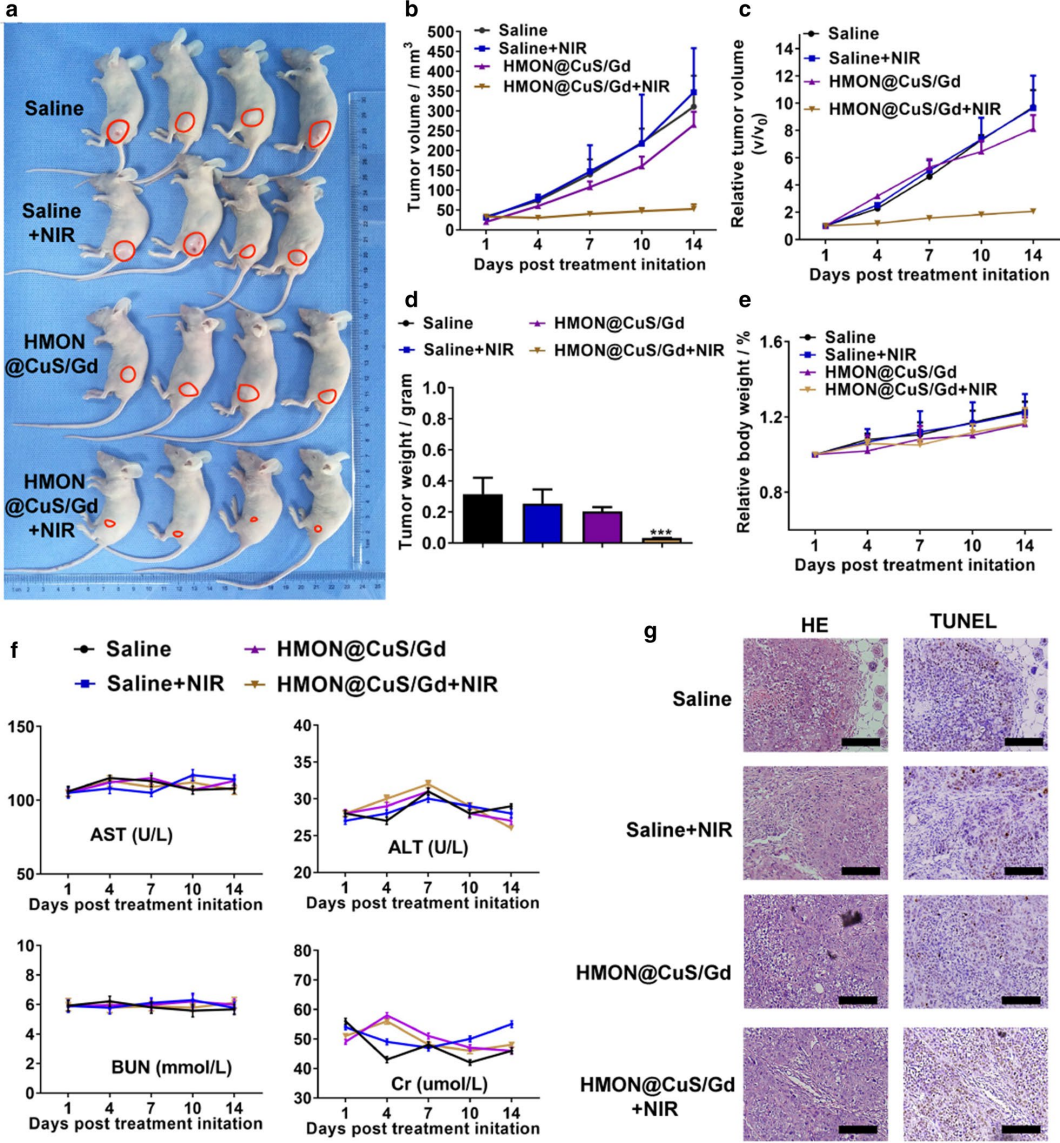
**a** Images of tumors derived from mice treated with saline, saline plus NIR, HMON@CuS/Gd and HMON@CuS/Gd plus NIR. **b**, **c** Tumor volumes and relative tumor volumes of HGC-27 tumors isolated from mice. **d** Tumor weights of HGC-27 tumors isolated from mice. **e** Relative body weights of HGC-27 tumor-bearing mice. **f** Biochemical assay results of the sera collected from mice. AST, aspartate aminotransferase; ALT, alanine aminotransferase; BUN, blood urea nitrogen; Cr, creatinine. **g** Histopathological analysis of tumors isolated from mice. Data are shown as mean ± SD, n = 4. Scale bar: 100 μm. *** indicates *P *< 0.001

To further analyze the antitumor effects of HMON@CuS/Gd NPs in vivo, we constructed tumor-bearing mice. Then, we randomly divided these mice into four groups, treating them with saline (negative control), saline plus NIR irradiation, HMON@CuS/Gd and HMON@CuS/Gd plus NIR irradiation. Fourteen-days’ post-treatment, we found that the relative tumor volume showed no significant difference between the saline group and saline plus NIR group, which meant that NIR itself could not inhibit tumor growth (Fig. 7a–d). Furthermore, inhibited tumor growth rate in the HMON@CuS/Gd plus NIR irradiation group was approximately 80% compared to control group, while free HMON@CuS/Gd induced no significant changes (Fig. 7a–d). We also observed the body-weight-change in these four groups, while no body-weight-loss was found (Fig. 7e). The blood serum samples were sent for the biochemical analysis of clinically relevant indicators (aspartate aminotransferase and alanine aminotransferase) for the liver and (blood urea nitrogen and creatinine) for the kidneys [47]. No significant differences were observed in free NIR, free HMON@CuS/Gd and HMON@CuS/Gd plus NIR groups, compared to saline control groups, indicating that no damage happened in livers or kidneys (Fig. 7f). Therefore, these results indicated that the photo-thermal therapy induced by HMON@CuS/Gd NPs could efficiently kill tumor cells with less side effect, indicating HMON@CuS/Gd NPs have a promising application for GC treatment.

The isolated issues were further sent for histological detection, while H&E staining (Fig. 7g) further confirmed the collected specimen were tumors. TUNEL termed as “Terminal deoxynucleotidyl transferase dUTP nick end labeling”, which is a classic marker to detect DNA fragmentation in apoptotic tumor cells [56]. Interestingly, we found that expression of TUNEL was upregulated in HMON@CuS/Gd plus NIR group, while no evident changes were found in other groups, compared with saline group (Fig. 7g; Additional file 1: Figure S21). Consequently, the results of IHC consistently highlight the superiority of HMON@CuS/Gd plus NIR irradiation on anti-tumor proliferation, since this group showed highest expression of TUNEL.

## Conclusion

In this work, we successfully constructed biocompatible and biodegradable Gd doped HMON decorated by CuS NPs (called HMON@CuS/Gd). The as-prepared NPs exhibited high photo-thermal conversion efficiency (82.4%) and ROS generation ability. Differently from the reported HMON@CuS NPs, it was found that hyperthermia might not be the only factor of the cell apoptosis, while ROS induced by PDT also plays an important role. PDT induced ROS would attack MTPs, promote mitoROS production and finally induced mitochondrial damage. Meanwhile, mitochondrial damage again changed anti/pro-apoptotic proteins, opened mPTP, released Cyto C into cytosol, and finally activated caspase-9/caspase-3-depended cell apoptosis pathway. In vivo data showed that HMON@CuS/Gd could serve as a nanoplatform for fluorescence/MRI/IRT triple modal imaging guided photo-therapy at cancer region, inhibit the growth of GC cells without evident systemic toxicity. Taken together, HMON@CuS/Gd could serve as a promising multifunctional nanotheranostic platform through inducing mitochondrial damage on GC.

## **Supplementary information**

**Additional file 1.** Additional Figures S1–S21.

## Data Availability

The datasets used during the present study are available from the corresponding author upon reasonable request.

## Abbreviations

HMON: Hollow mesoporous organosilica nanotheranostics
HMON@CuS: CuS-modified hollow mesoporous organosilica nanoparticles
NIR: Near infrared
PTT: Photo-thermal therapy
PDT: Photo-dynamic therapy
GC: Gastric cancer
HMON@CuS/Gd: Gd doped HMON decorated by CuS nanocrystals
GSH: l-Glutathione
ROS: Reactive oxygen species
MTPs: Mitochondrial transmembrane potentials
MitoROS: Mitochondrial reactive oxygen species
mPTP: Mitochondrial permeability transition pore
FL: Fluorescence
MRI: Magnetic resonance imaging
IRT: Infrared thermal
mitoDNA: Mitochondrial DNA
Cyto C: Cytochrome C
PT: Photo-therapy
CTAC: Cetyltrimethylammonium chloride solution
TEA: Triethanolamine
TEOS: Tetraethoxysilane
MPTES: Sodium citrate (3-mercaptopropyl)-trimethoxysilane
BTES: Bis[3-(triethoxysilyl)propyl]tetrasulfide
CuCl_2_: Copper chloridedihydrate
Na_2_S·9H_2_O: Sodium sulfide nonahydrate
GdCl_3_·6H_2_O: Gadolinium chloride hexahydrate
FBS: Fetal bovine serum
ATCC: American Type Culture Collection
ddH_2_O: Deionized water
FT-IR: Fourier transform infrared spectroscopy
XRD: X-ray diffraction
XPS: X-ray photoelectron spectroscopy
CLSM: Confocal laser scanning microscope
BSA: Bovine serum albumin
FITC: Fluorescein isothiocyanate
IHC: Immunohistochemistry
SD: Standard deviation
IC50: Half maximal inhibitory concentration
FACS: Flow cytometry analysis
NAC: *N*-Acetyl-l-cysteine
EPR: Enhanced permeability and retention
