# Supplementary material for: Biodegradable hollow mesoporous organosilica nanotheranostics (HMON) for multi-mode imaging and mild photo-therapeutic-induced mitochondrial damage on gastric cancer
Source: J Nanobiotechnology. 2020 Jul 20;18:99. doi: 10.1186/s12951-020-00653-y (PMC7370480; doi:10.1186/s12951-020-00653-y)
Supplement: Supplementary file 1 — Additional file 1. Additional Figures S1–S21. [file 12951_2020_653_MOESM1_ESM.doc]

**Running Title: Biodegradable hollow mesoporous organosilica nanotheranostics (HMON) for** **multi-mode imaging and mild phototherapeutic-induced mitochondrial fusion on gastric cancer**

Additional Material

***Results***

***
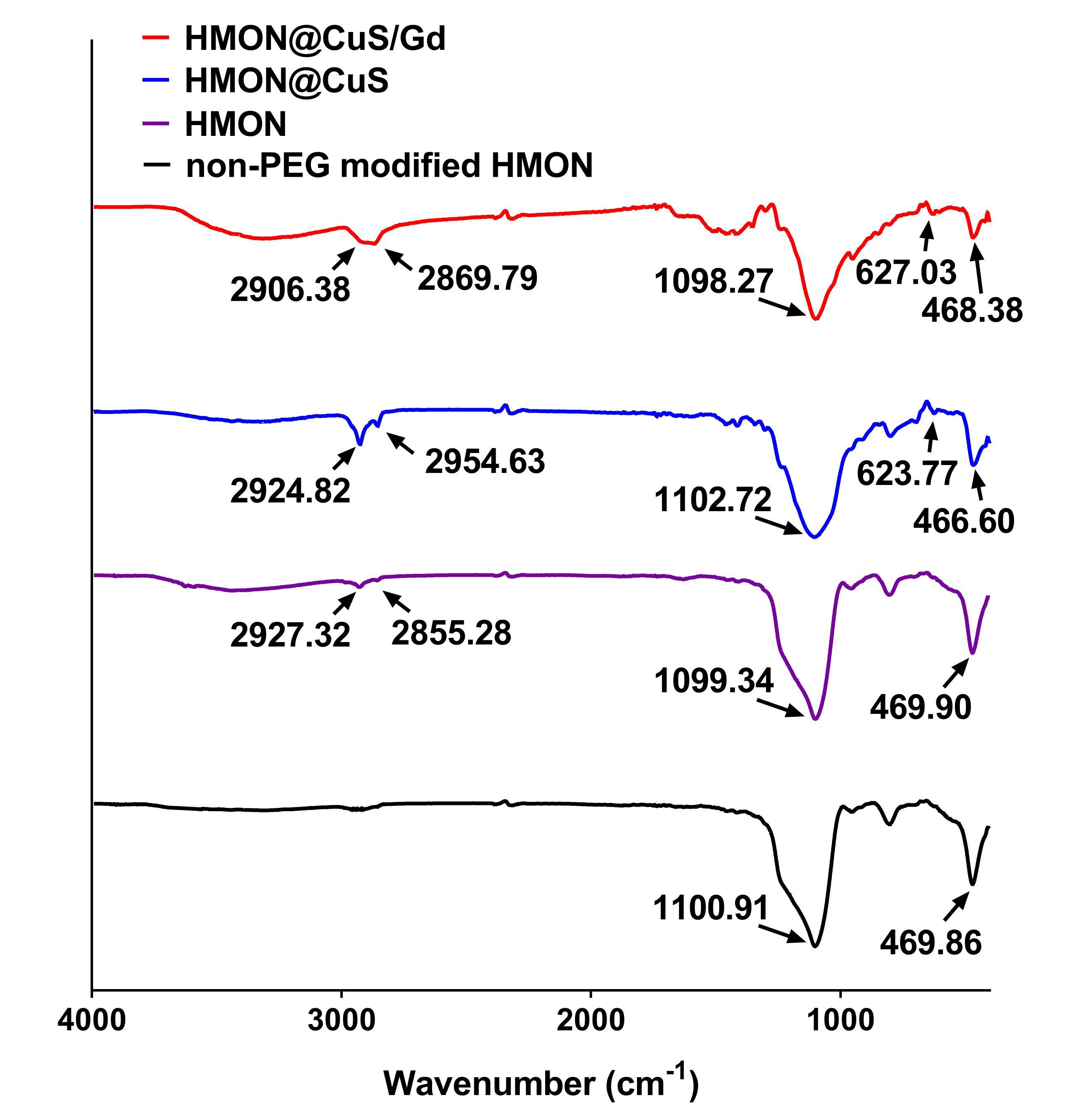
***

Figure S1. The FT-IR of non-PEG modified HMON, and PEG modified HMON, HMON@CuS and HMON@CuS/Gd nanoparticles.


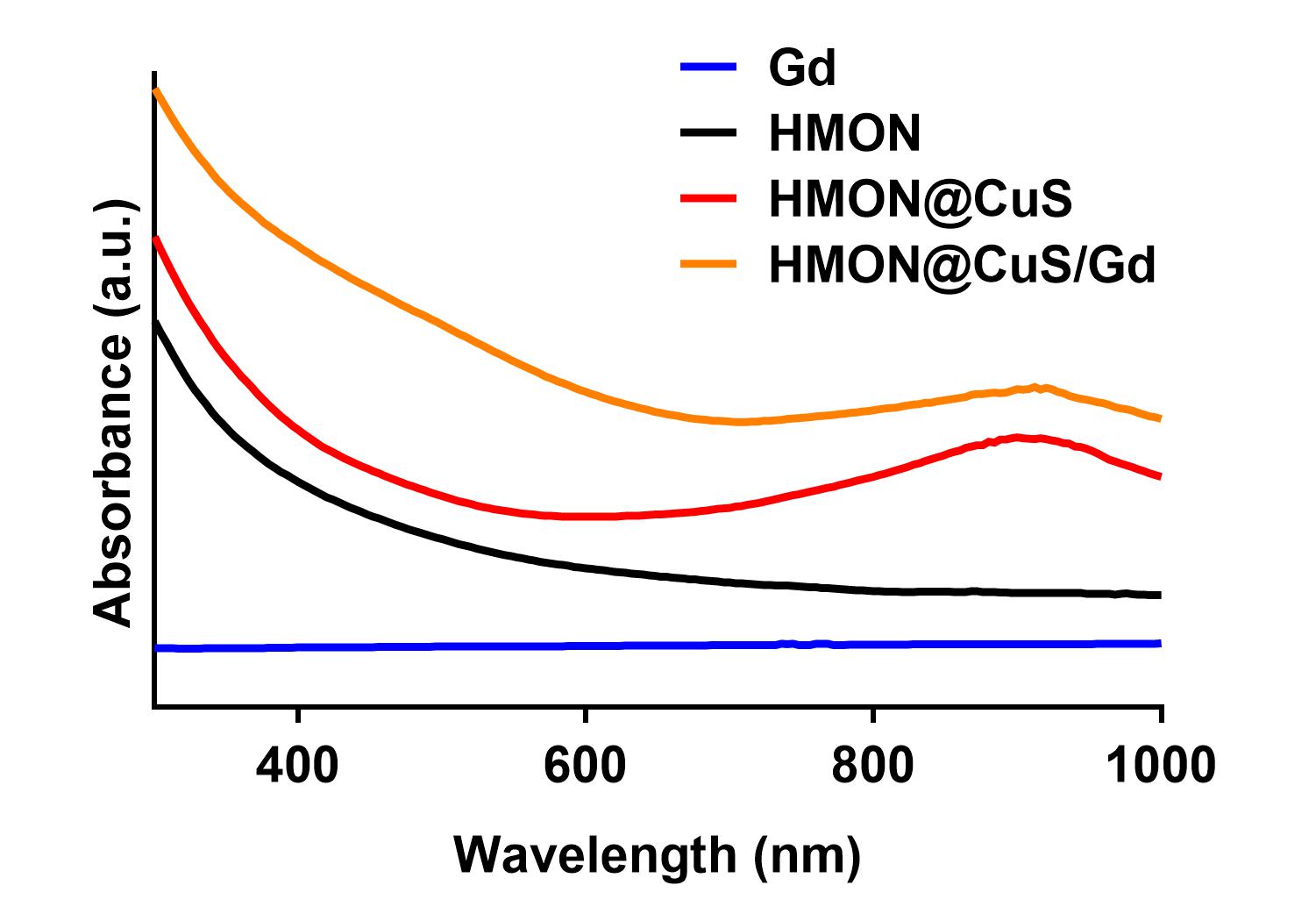


Figure S2. The UV−vis absorption spectrum of Gd, HMON, HMON@CuS and HMON@CuS/Gd nanoparticles.


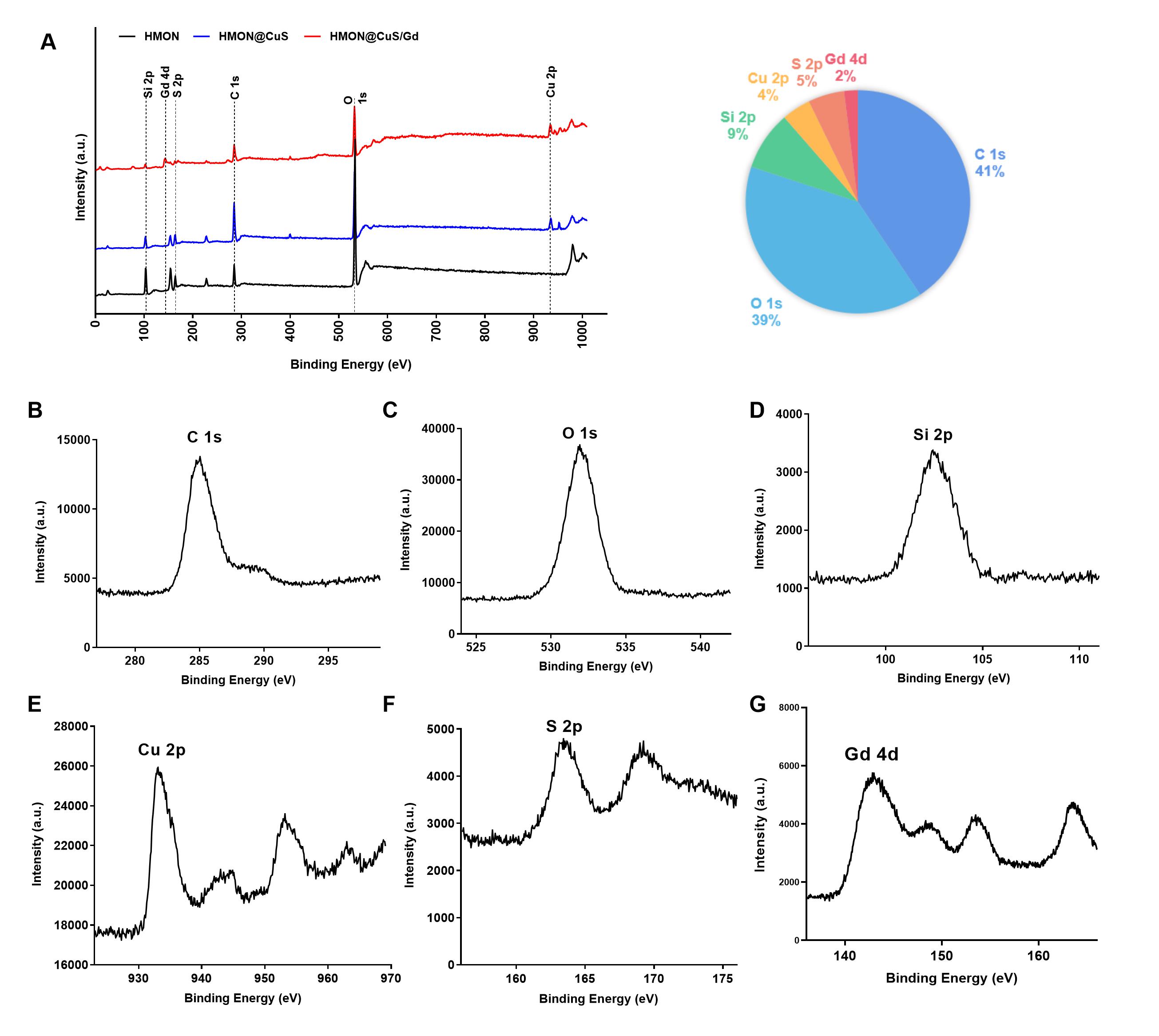


Figure S3. (A) The XPS analysis of HMON, HMON@CuS and HMON@CuS/Gd, and the content analysis of C, O, Si, Cu, S and Gd elements in HMON@CuS/Gd. (B-G) The XPS analysis of C, O, Si, Cu, S and Gd elements in HMON@CuS/Gd.


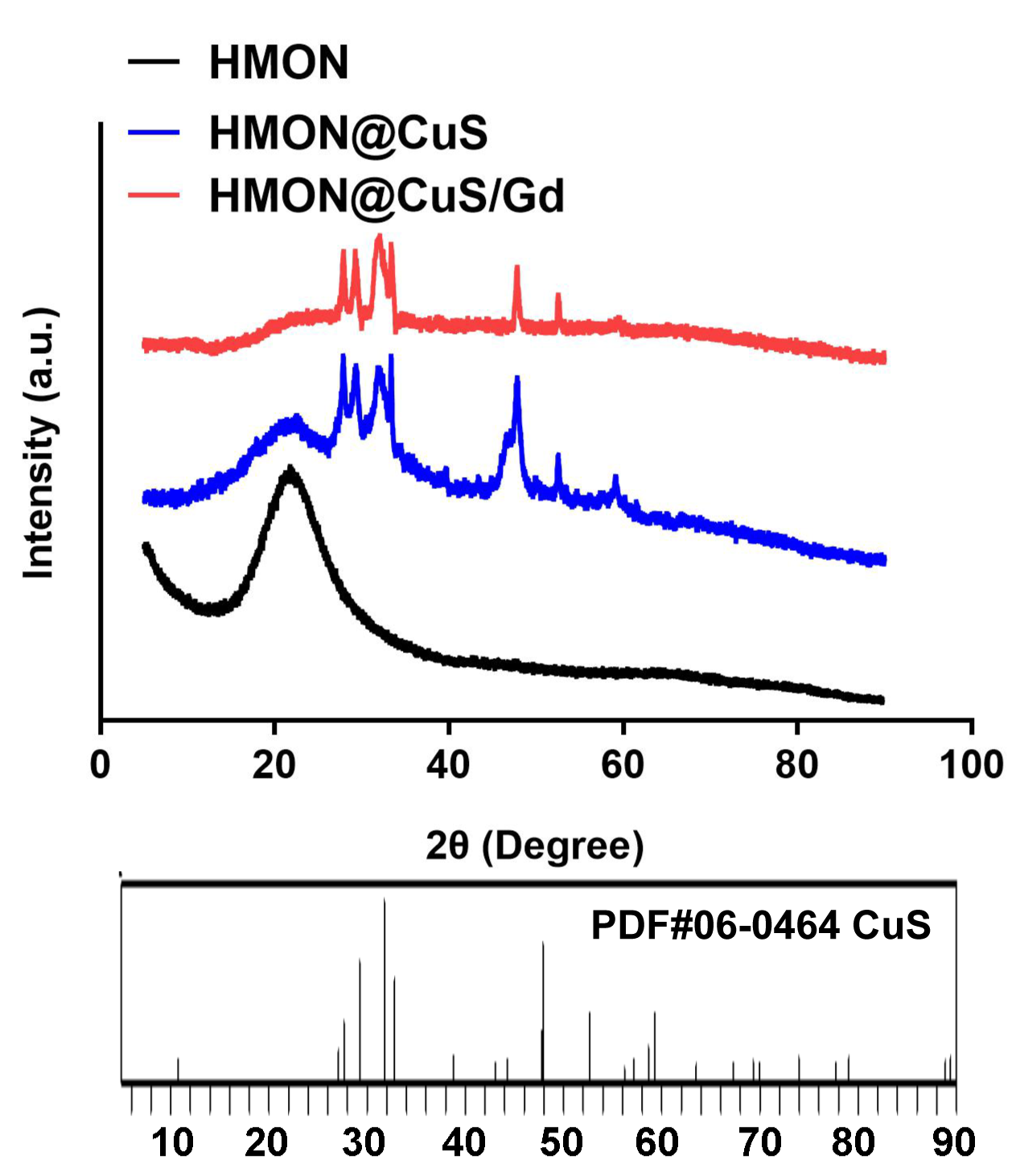


Figure S4. The XRD analysis of HMON, HMON@CuS and HMON@CuS/Gd nanoparticles.


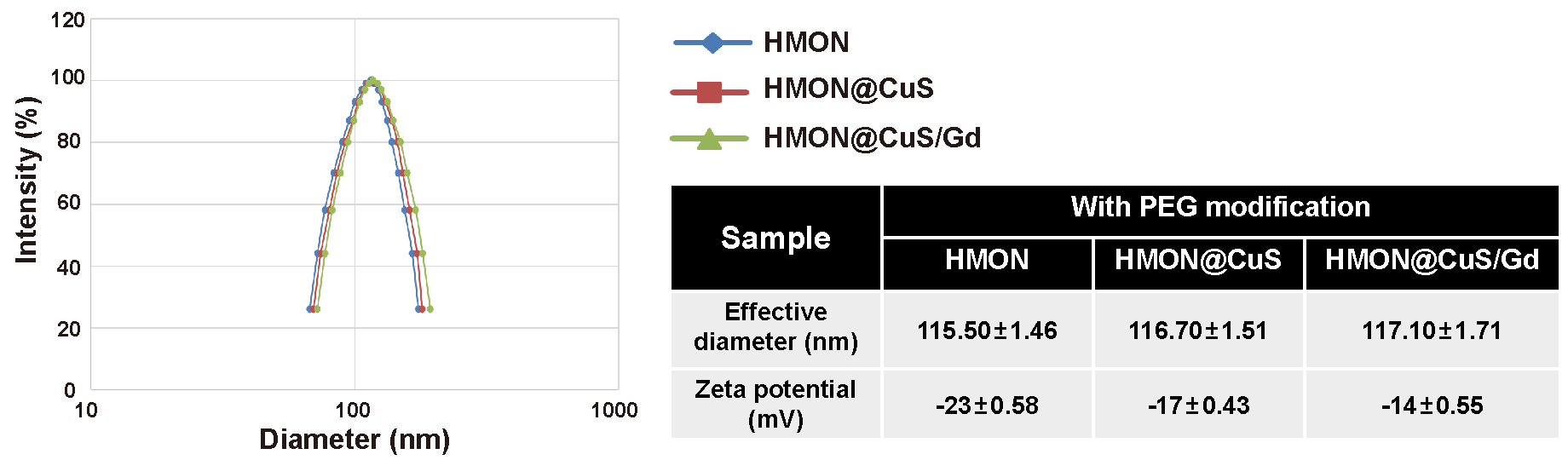
 Figure S5. The size distribution, diameters and zeta potential of HMON, HMON@CuS and HMON@CuS/Gd nanoparticles (with PEG modification).


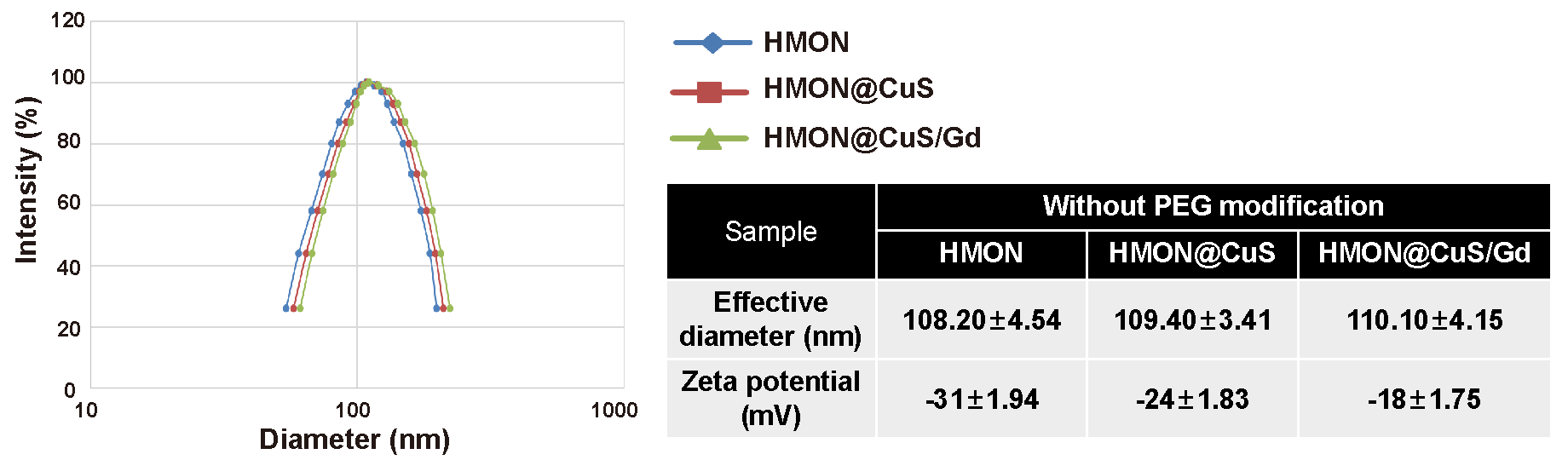


Figure S6. The size distribution, diameters and zeta potential of HMON, HMON@CuS and HMON@CuS/Gd nanoparticles (without PEG modification).


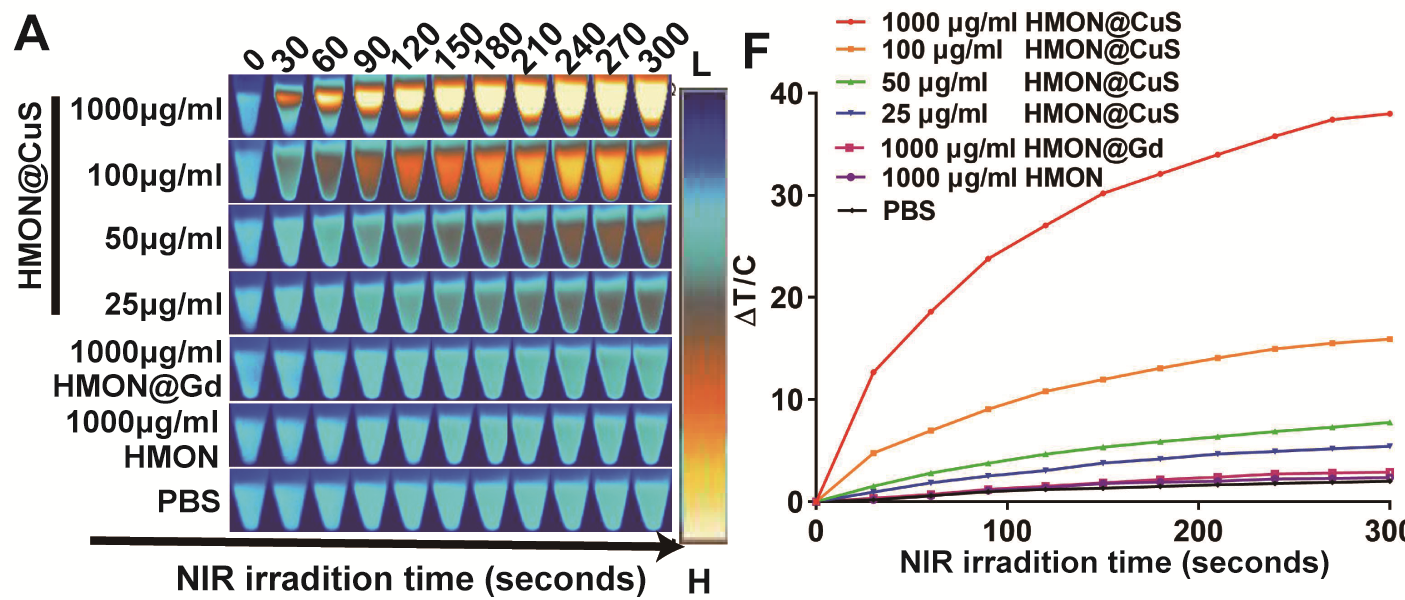


Figure S7. The temperature increase curve induced by different concentrations of HMON@CuS solutions, HMON@Gd, HMON and PBS under NIR irradiation (0.8 W/cm^2^, 5 mins)


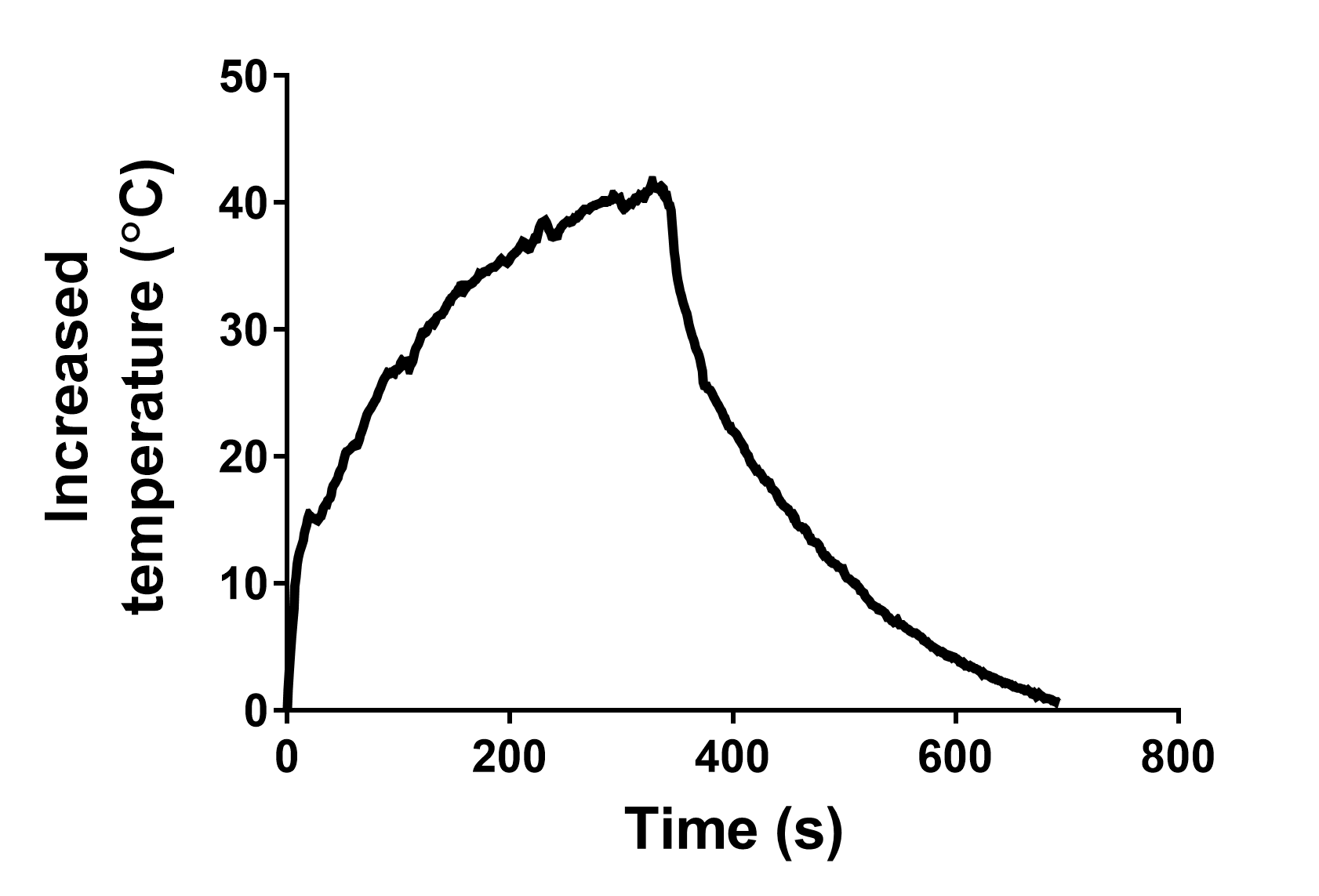


Figure S8. Photothermal performance of HMON@CuS/Gd nanoparticles at a concentration of 1000µg/mL under NIR irradiation, with the laser cut off when the temperature became stable.

**
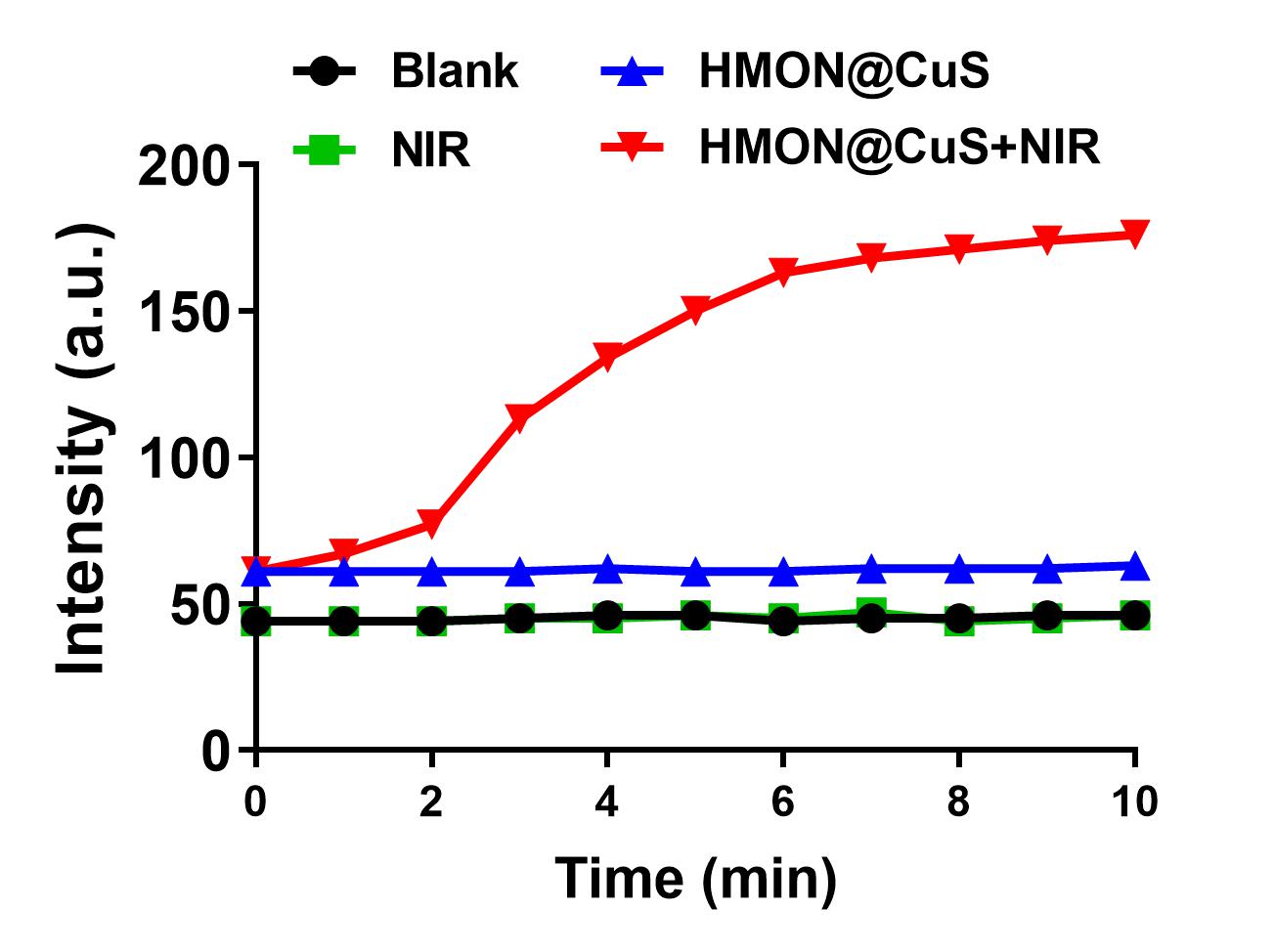
**

Figure S9. The production of singlet oxygen by HMON@CuS NPs with or without NIR irradiation (0.8 W/cm^2^, 5 mins).

**
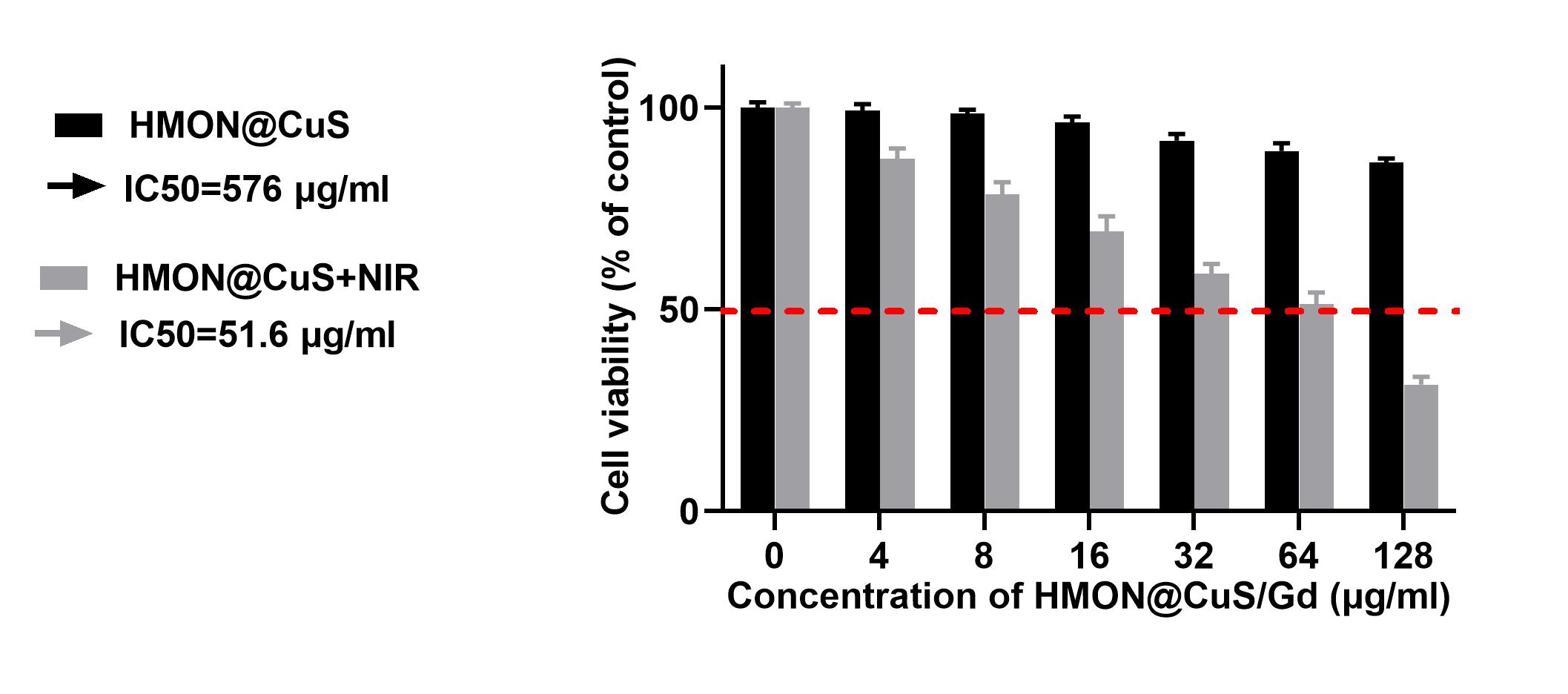
**

Figure S10. Cell viabilities of HMON@CuS treated HGC-27 cells, with or without NIR irradiation (0.8 W/cm^2^, 5 mins), using CCK-8 assay.


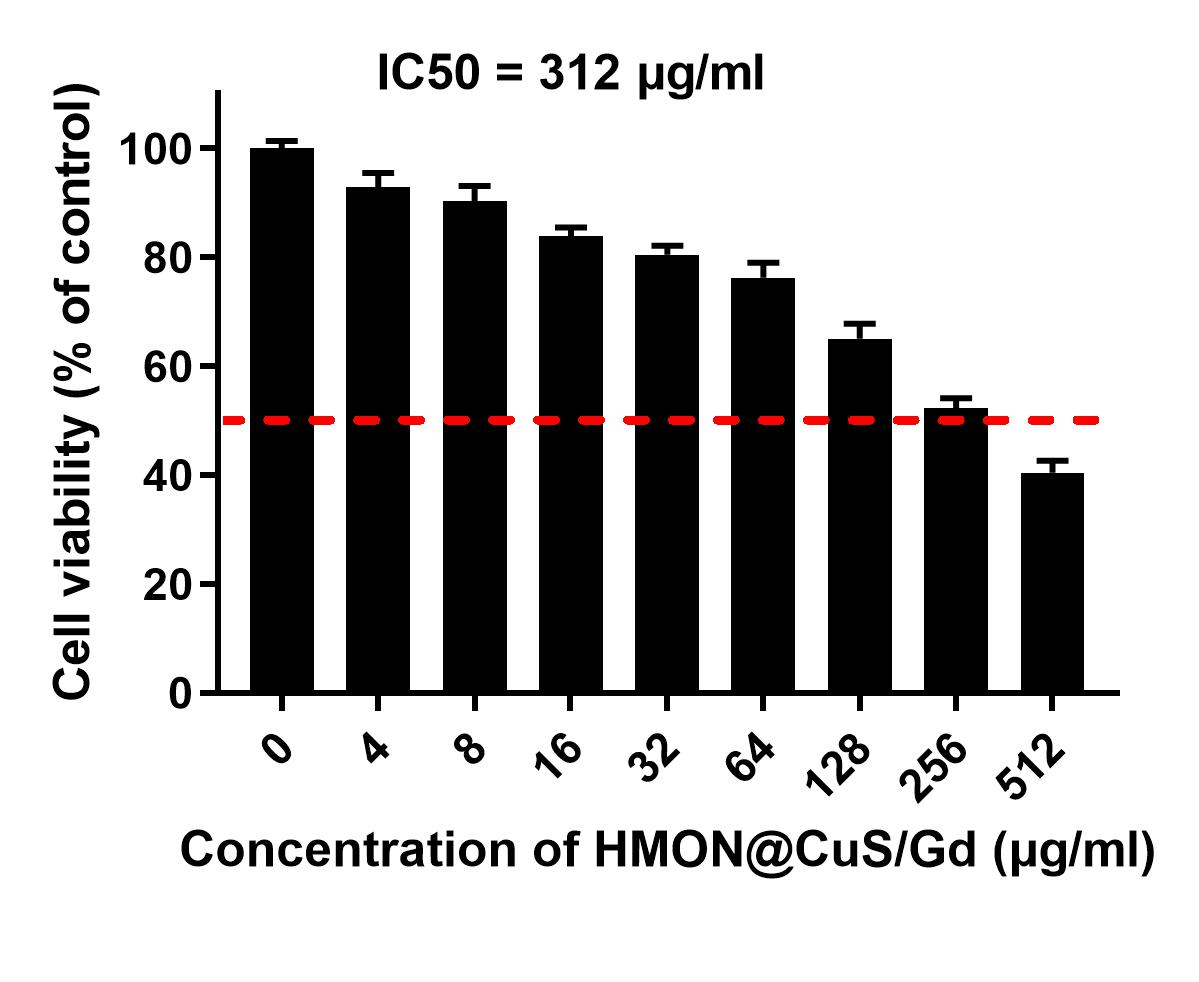


Figure S11. Cell viabilities of HGC-27 cells, after treated with HMON@CuS/Gd (without PEG modification), using CCK-8 assay.

**
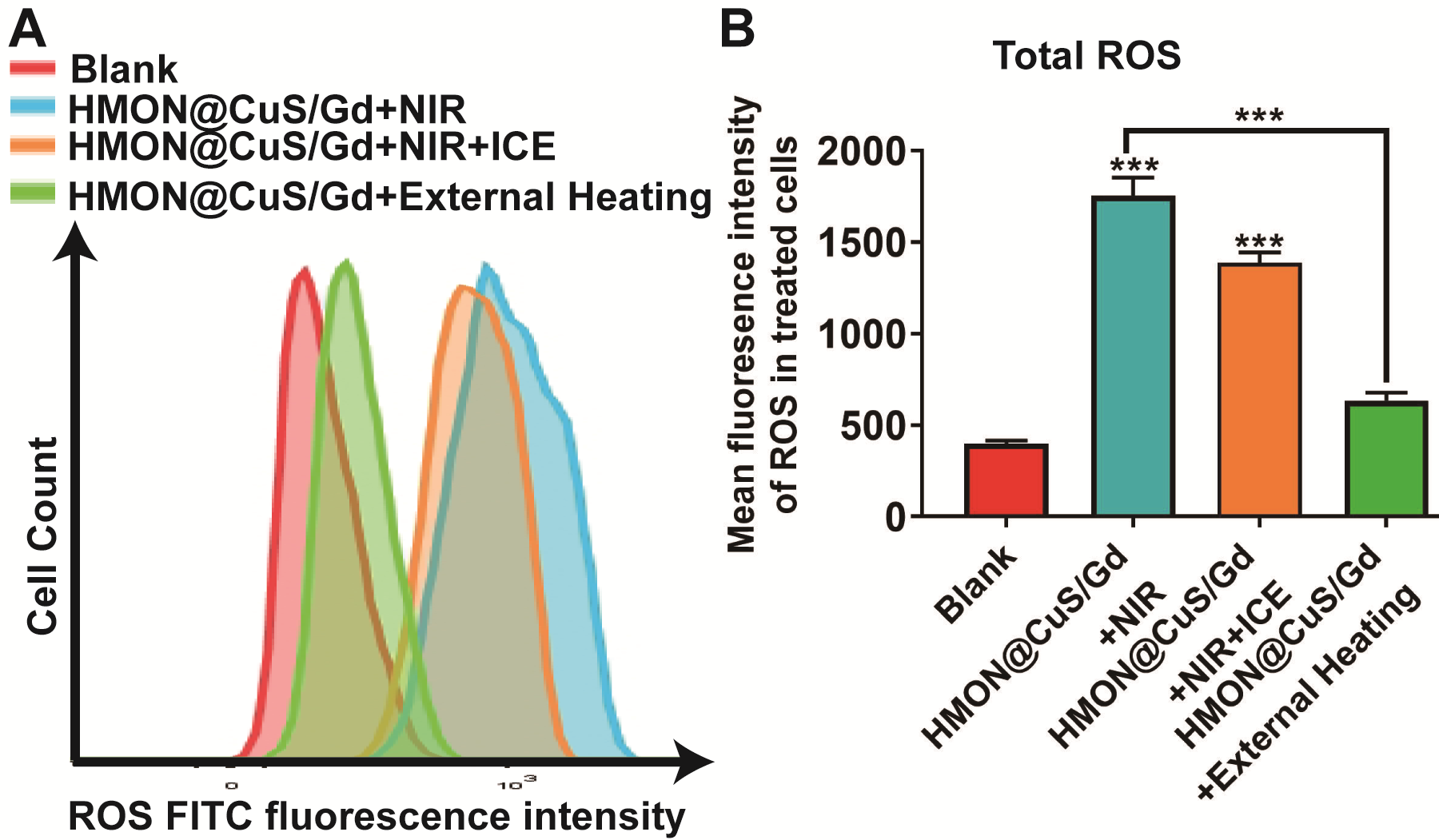
**

Figure S12. (A) Total ROS images of HGC-27 cells after treated with single HMON@CuS/Gd plus NIR, HMON@CuS/Gd plus NIR on ice or HMON@CuS/Gd plus external heating, detected by flow cytometry. (B) Statistical graph of ROS content in HGC-27 cells. Data is shown as the mean ± SD, n=5. *** indicates *P*＜0.001.


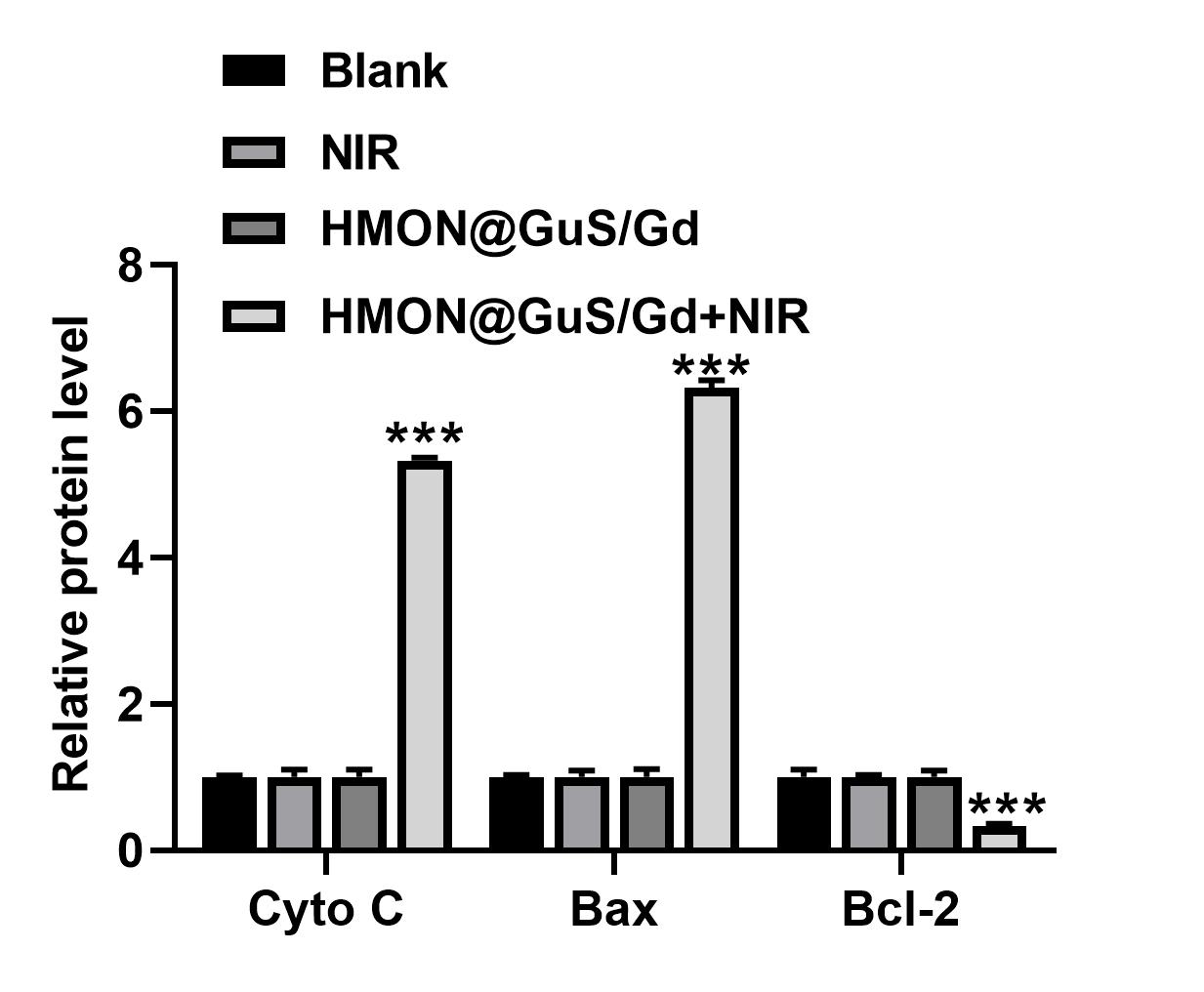


Figure S13. The statistical gray value graph of Cyto C, Bax and Bcl-2 proteins’ expression in HGC-27 cells after treated with HMON@CuS/Gd, sing NIR and HMON@CuS/Gd plus NIR, using Image J software. Data is shown as the mean ± SD, n=3. *** indicates *P*＜0.001.


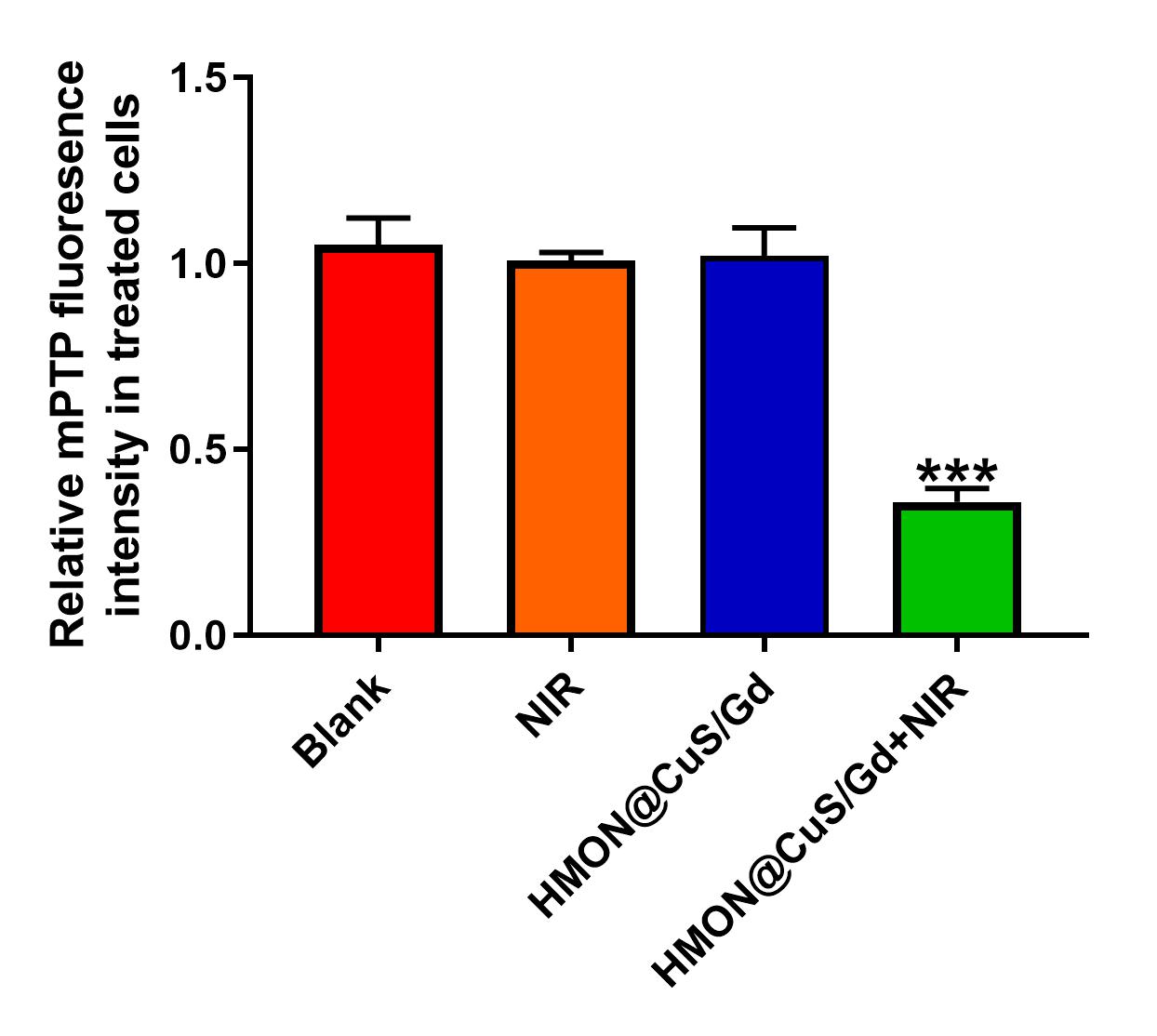


Figure S14. The statistical mPTP fluorescence intensity in HGC-27 cells after treated with HMON@CuS/Gd, sing NIR and HMON@CuS/Gd plus NIR. Data is shown as the mean ± SD, n=3. *** indicates *P*＜0.001.


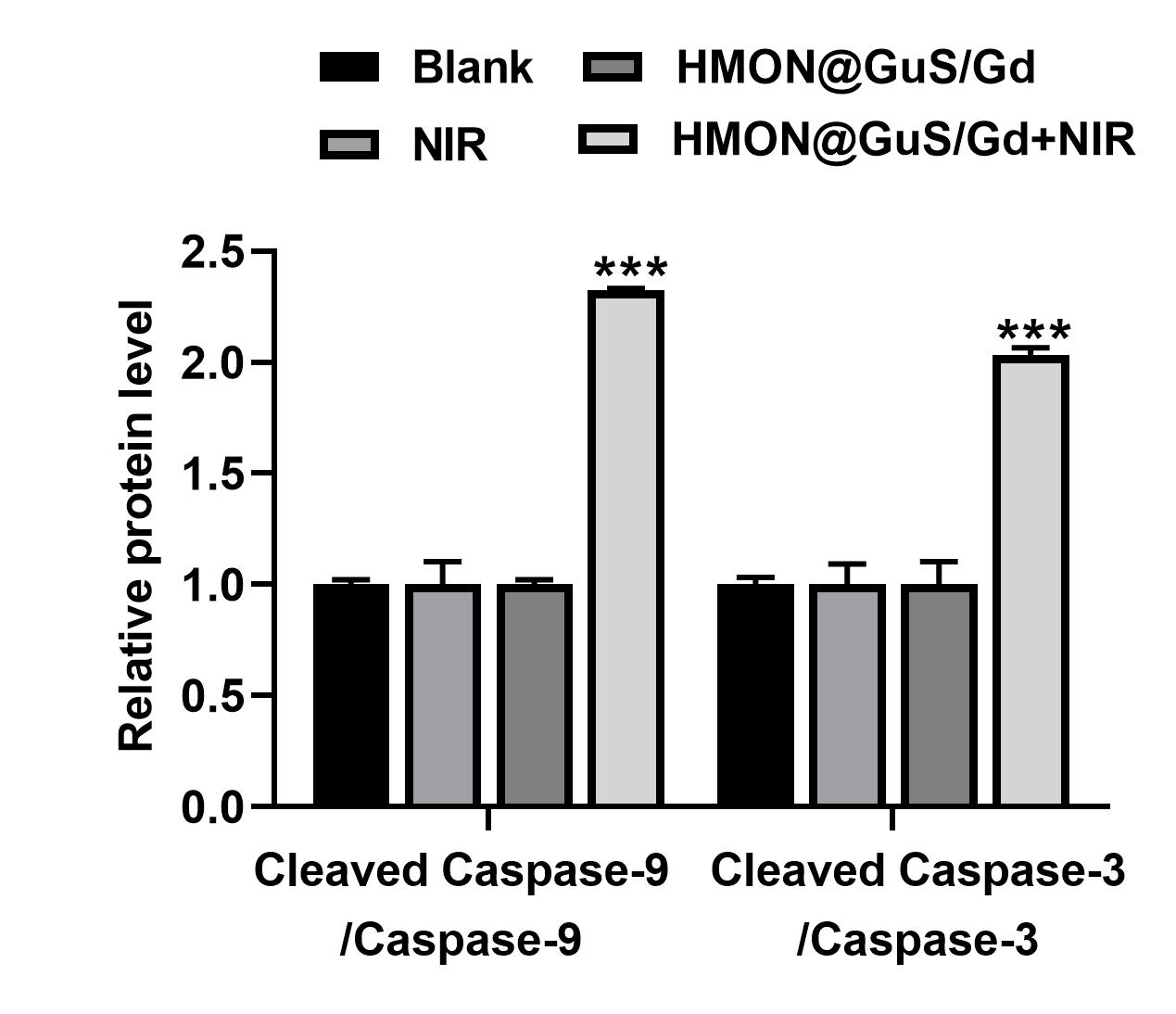


Figure S15. The statistical gray value graph of Cleaved Caspase-9/ Caspase-9 and Cleaved Caspase-3/ Caspase-3 in HGC-27 cells after treated with HMON@CuS/Gd, sing NIR and HMON@CuS/Gd plus NIR, using Image J software. Data is shown as the mean ± SD, n=3. *** indicates *P*＜0.001.


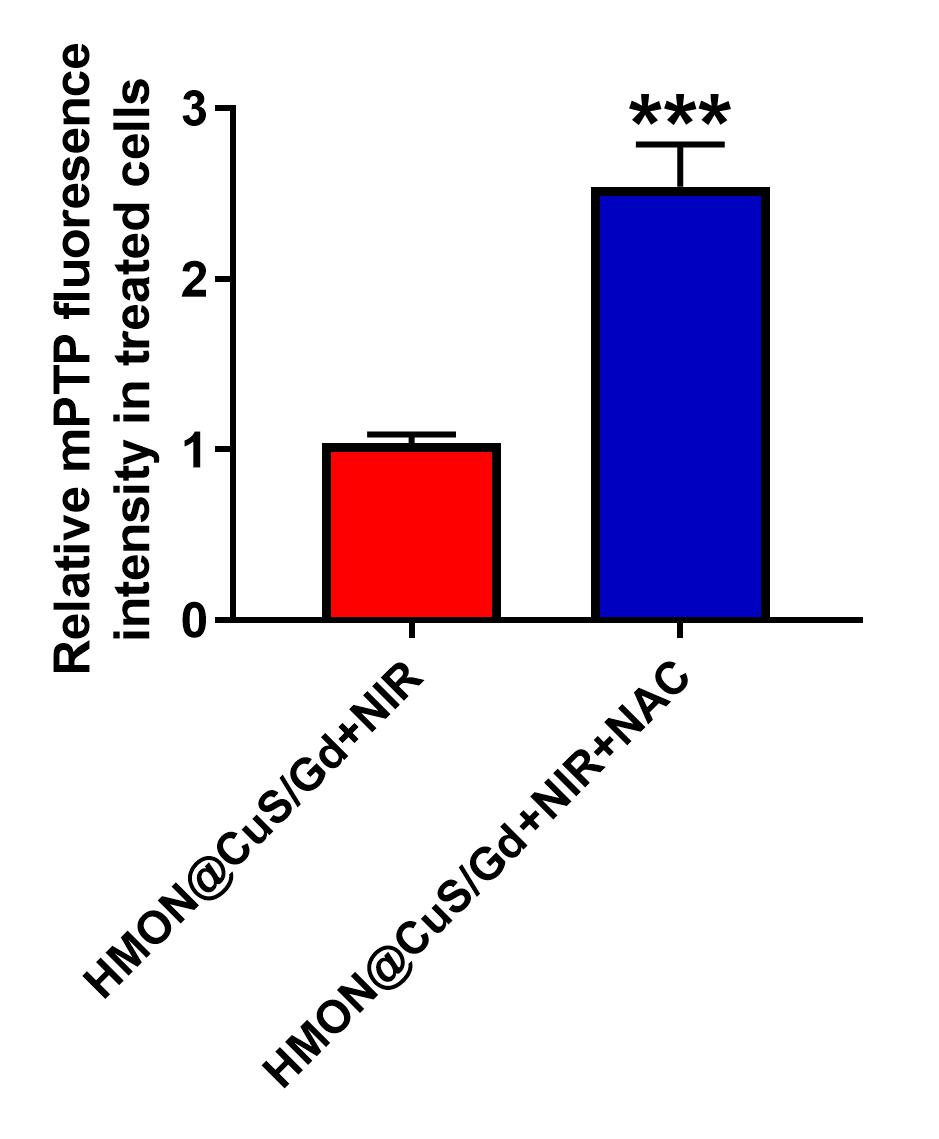


Figure S16. The statistical mPTP fluorescence intensity in HGC-27 cells after treated with HMON@CuS/Gd plus NIR, with or without the addition of NAC. Data is shown as the mean ± SD, n=3. *** indicates *P*＜0.001.


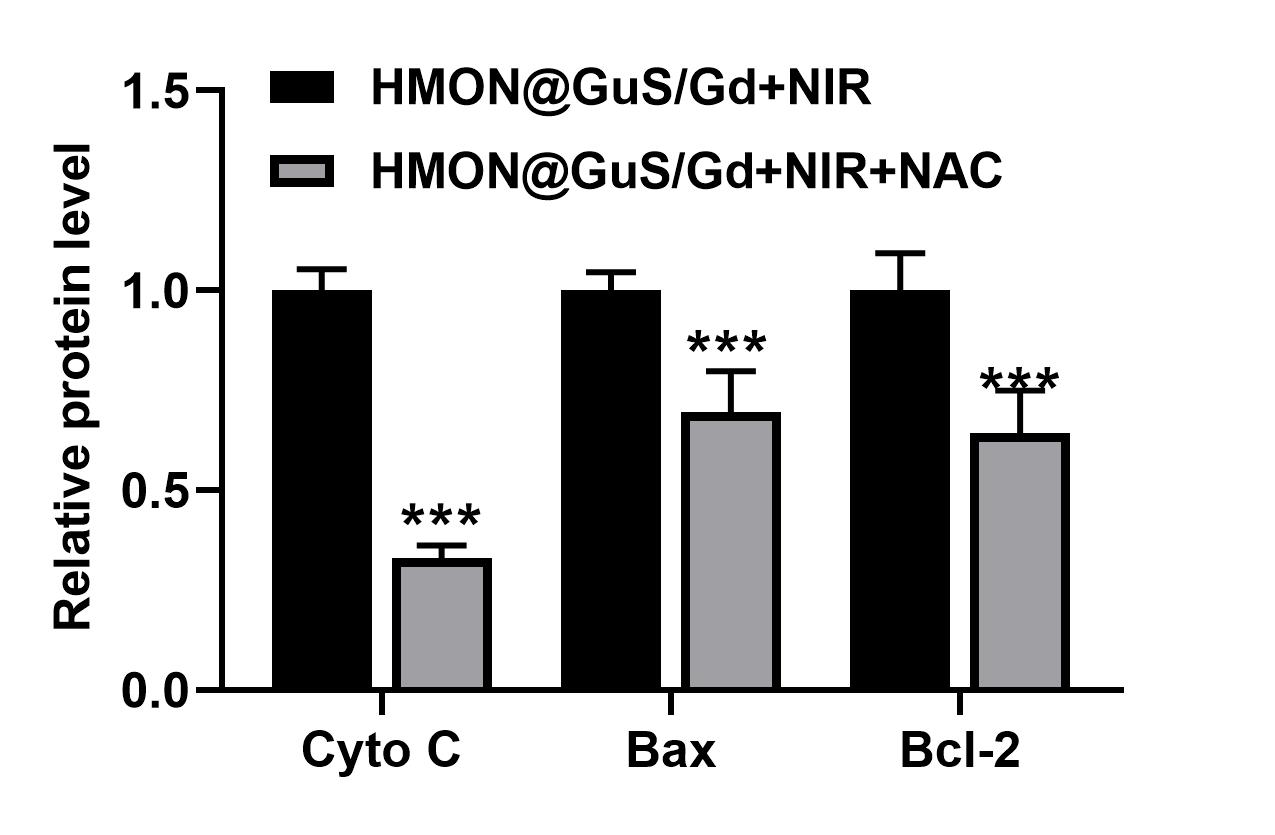


Figure S17. The statistical gray value graph of Cyto C, Bax and Bcl-2 proteins’ expression in HGC-27 cells after treated with HMON@CuS/Gd plus NIR, with or without the addition of NAC, using Image J software. Data is shown as the mean ± SD, n=3. *** indicates *P*＜0.001.


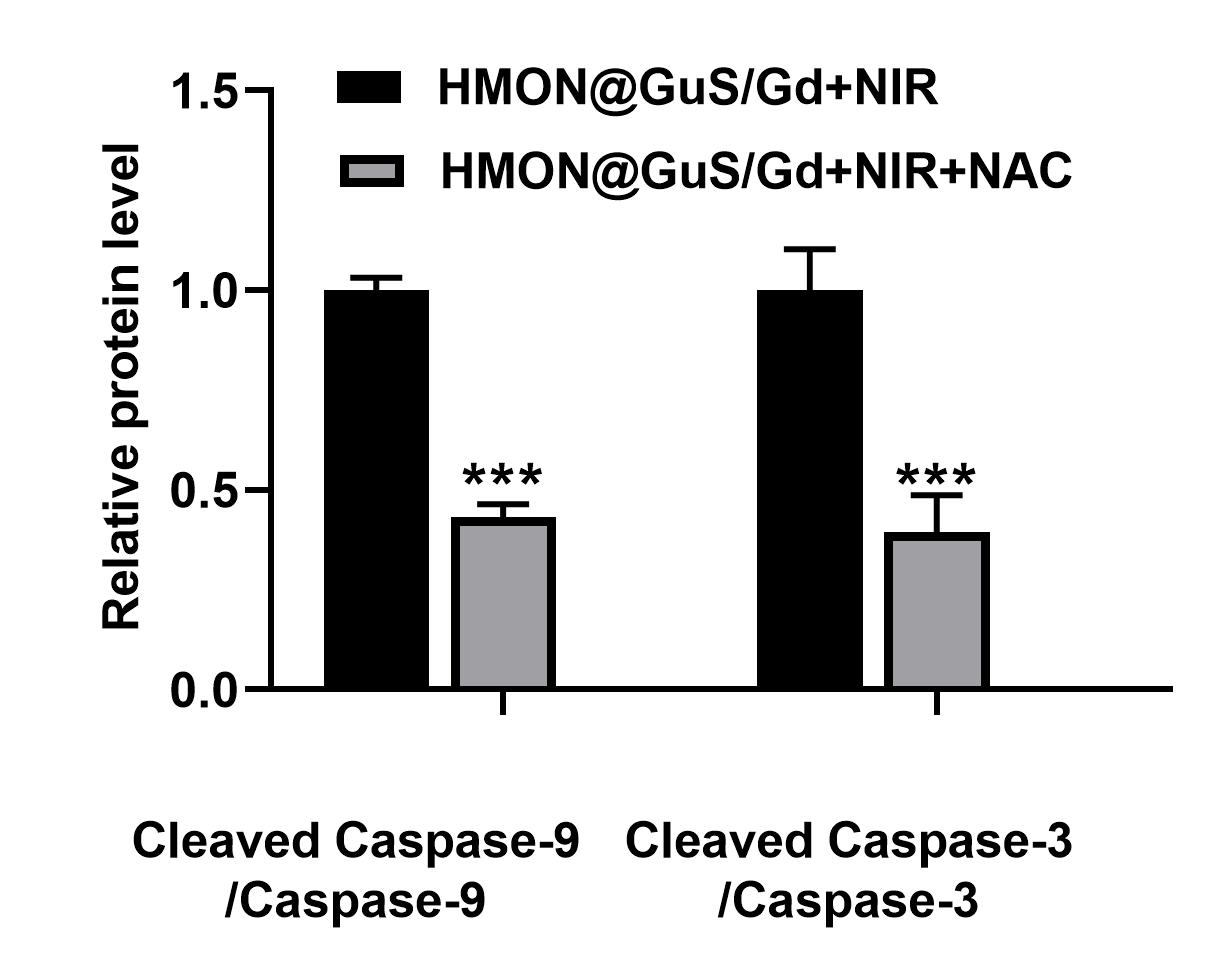


Figure S18. The statistical gray value graph of Cleaved Caspase-9/ Caspase-9 and Cleaved Caspase-3/ Caspase-3 in HGC-27 cells after treated with HMON@CuS/Gd plus NIR, with or without the addition of NAC, using Image J software.


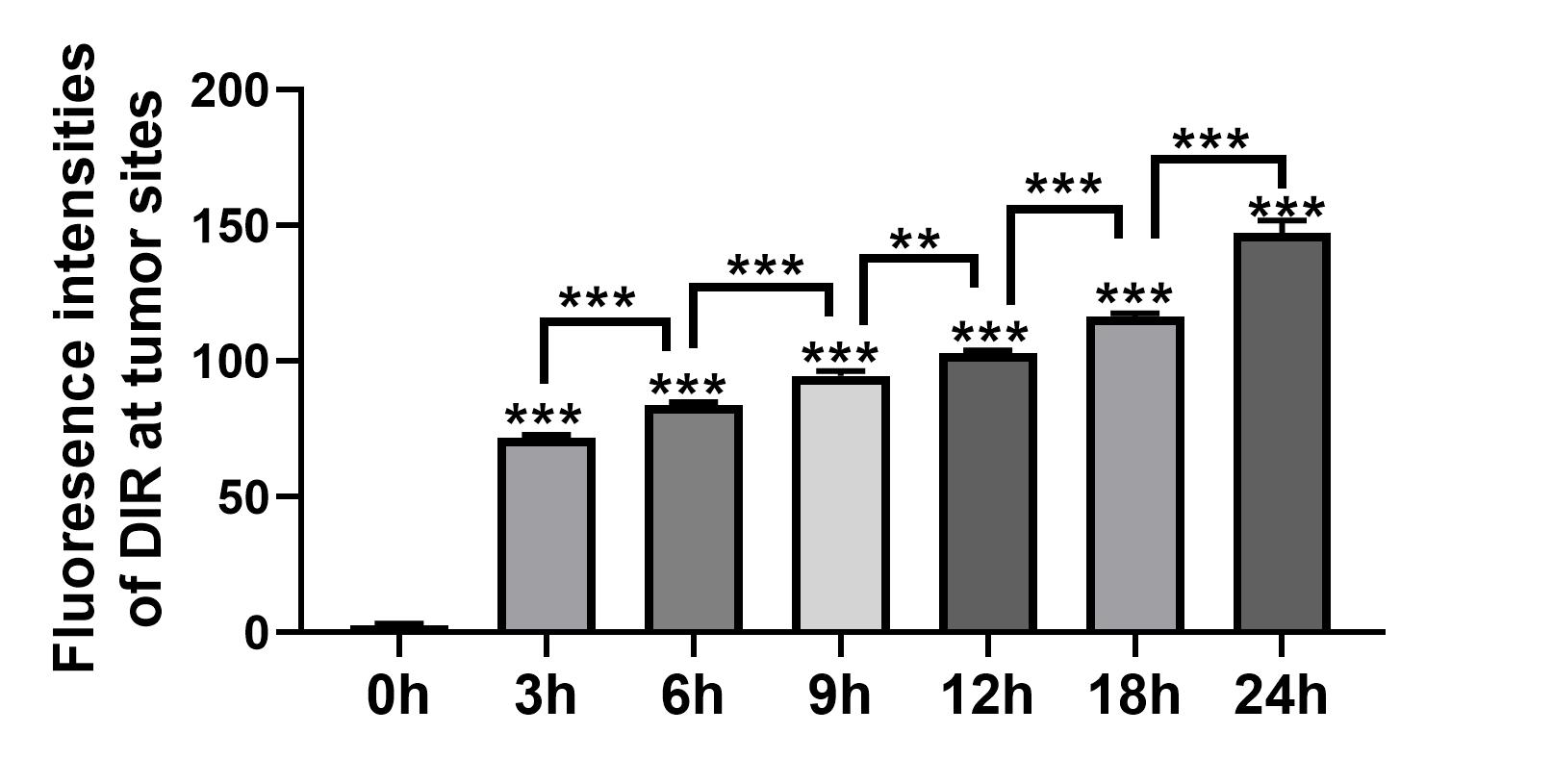


Figure S19. The statistical gray value graph of DIR in HGC-27 bearing mice, at 0, 3, 6, 9, 12, 18 and 24 timepoints, using Image J software. Data is shown as the mean ± SD, n=3.


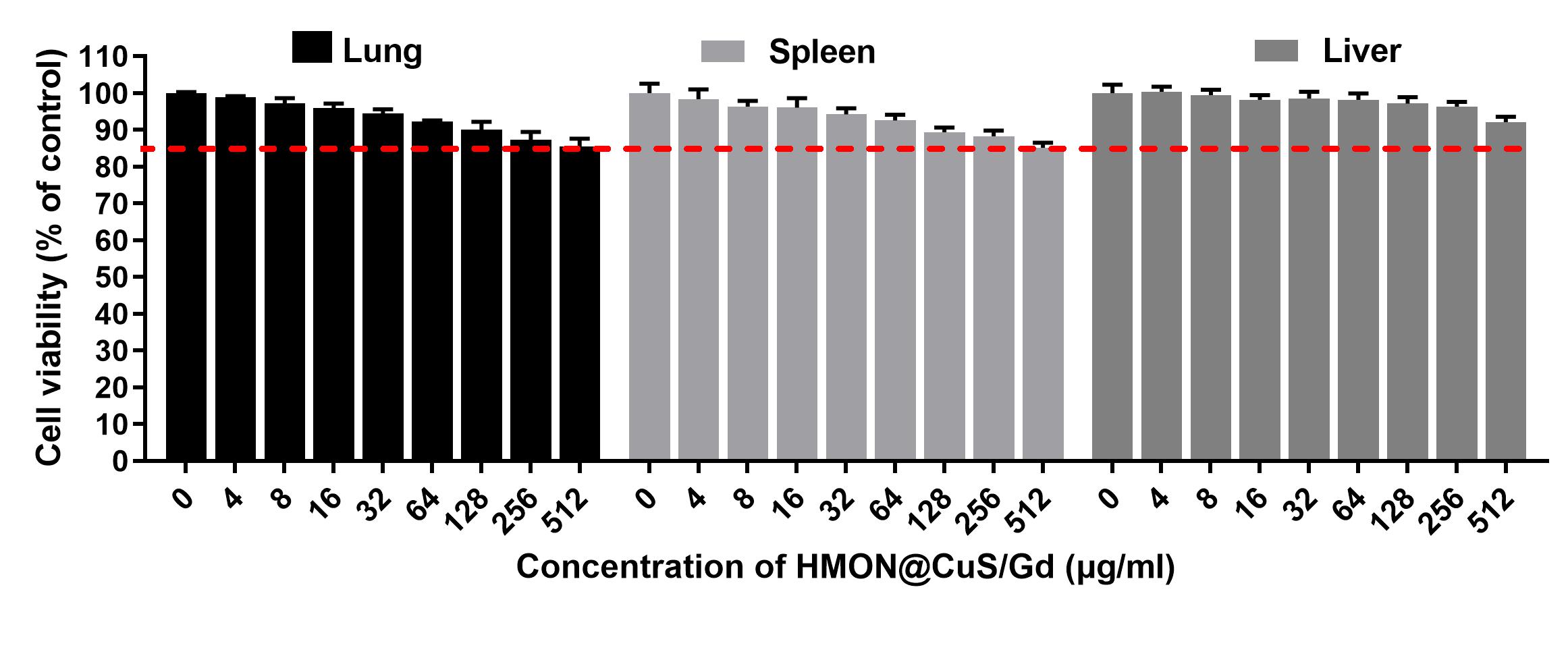


Figure S20. Cell viabilities of human lung normal epithelial cells (BEAS-2B cells), primary spleen cells of mice (spleen cells) and human liver normal epithelial cells (LO2 cells), after treated with HMON@CuS/Gd (with PEG modification), using CCK-8 assay. Data is shown as the mean ± SD, n=5.


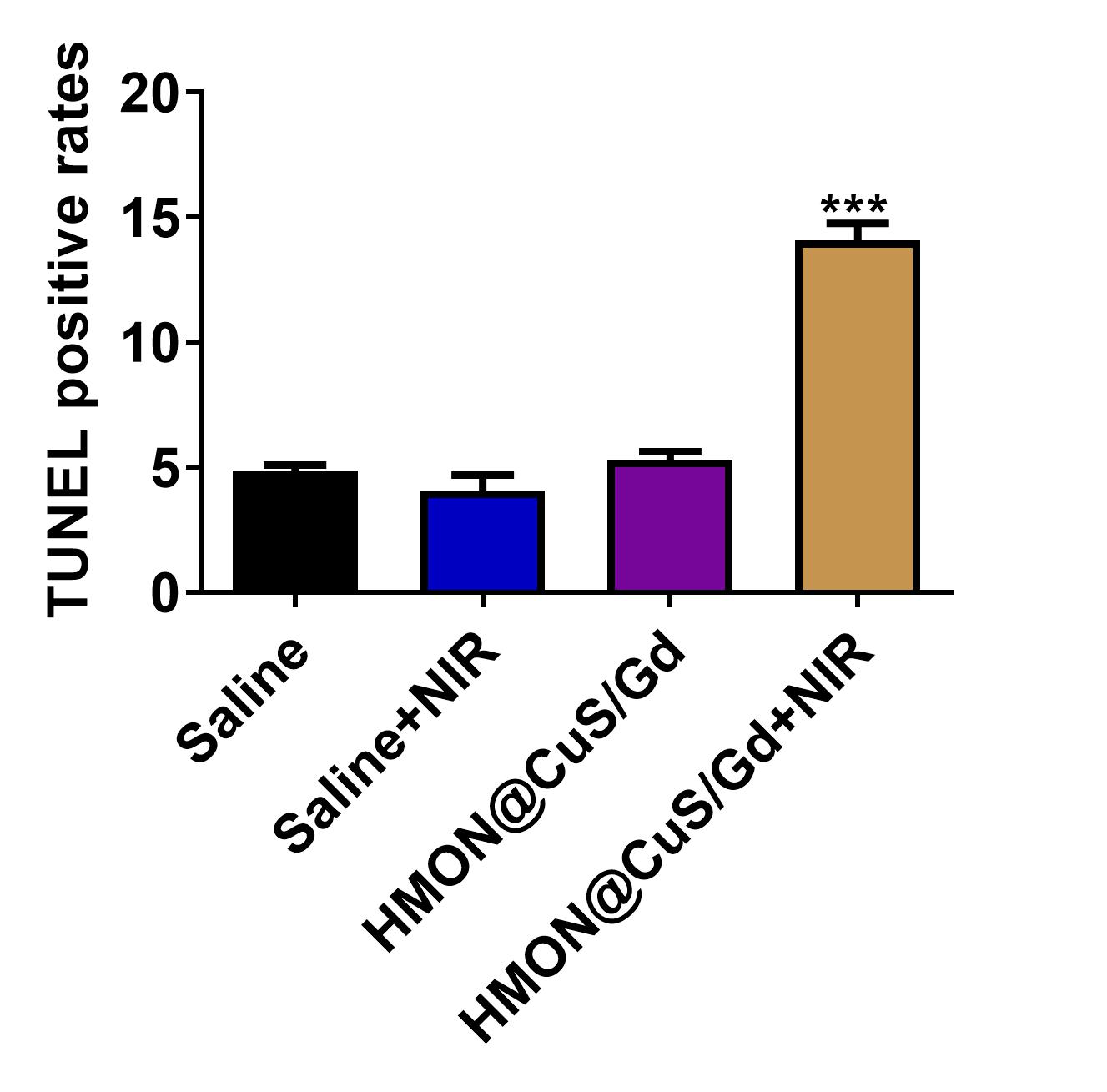


Figure S21. TUNEL positive rates in mice treated with saline, saline plus NIR, HMON@CuS/Gd and HMON@CuS/Gd plus NIR. Data is shown as the mean ± SD, n=4.
